# Metalign: efficient alignment-based metagenomic profiling via containment min hash

**DOI:** 10.1186/s13059-020-02159-0

**Published:** 2020-09-10

**Authors:** Nathan LaPierre, Mohammed Alser, Eleazar Eskin, David Koslicki, Serghei Mangul

**Affiliations:** 1 Department of Computer Science, University of California, Los Angeles, CA 90095 USA; 2 Department of Computer Science, ETH Zurich, Rämistrasse 101, CH-8092 Zurich, Switzerland; 3 Department of Computational Medicine, University of California, Los Angeles, CA 90095 USA; 4 Department of Human Genetics, University of California, Los Angeles, CA 90095 USA; 5 Department of Computer Science and Engineering, The Pennsylvania State University, University Park, PA USA; 6 Department of Biology, The Pennsylvania State University, University Park, PA USA; 7 Huck Institutes of the Life Sciences, The Pennsylvania State University, University Park,, PA USA; 8 Department of Clinical Pharmacy, University of Southern California, Los Angeles, CA 90089 USA

**Keywords:** Metagenomics, Abundance estimation, Profiling, Alignment

## Abstract

Metagenomic profiling, predicting the presence and relative abundances of microbes in a sample, is a critical first step in microbiome analysis. Alignment-based approaches are often considered accurate yet computationally infeasible. Here, we present a novel method, Metalign, that performs efficient and accurate alignment-based metagenomic profiling. We use a novel containment min hash approach to pre-filter the reference database prior to alignment and then process both uniquely aligned and multi-aligned reads to produce accurate abundance estimates. In performance evaluations on both real and simulated datasets, Metalign is the only method evaluated that maintained high performance and competitive running time across all datasets.

## Introduction

Microorganisms are ubiquitous in almost every natural setting, including soil [1], ocean water [2], and the human body [3], and they play critical roles in the functioning of each of these systems [4, 5]. Traditional culture-based analysis of these microbes is confounded by the presence of many microorganisms that cannot be cultured in standard laboratory settings [4, 6]. Further, analysis of lab-cultured organisms fails to capture the complex community dynamics in real microbial ecosystems [4]. The field of metagenomics, or the analysis of whole microbial genomes recovered directly from their host environment via high-throughput sequencing, is vital to understanding microbial communities and their functions [4, 5].

Predicting the presence and relative abundance of taxa in a metagenomic sample (referred to as “taxonomic profiling”) is one of the primary means of analyzing a metagenomic sample [7, 8]. In comparison with metagenomic assembly, profiling is computationally simpler and more effective at identifying low-abundance organisms [8]. Metagenomic profiles can be obtained through read classification (where individual reads are assigned to taxa or organisms) or via the closely related technique of read binning (grouping individual reads into putative single taxa or organism groups). However, due to the necessary step of inferring taxa from individual short reads, these strategies have been shown to produce less accurate taxonomic profiles [7].

A recent widely cited comprehensive benchmarking study, the Critical Assessment of Metagenome Interpretation (CAMI) [7], evaluated ten widely used profiling methods on a variety of simulated metagenomic datasets. Notably, the two profiling methods that performed best in precision (i.e., false-positive rate) were the two methods ranked lowest for recall (i.e., false-negative rate); similarly, the two best methods for recall were two of the three methods ranked lowest for precision [7]. Another major benchmarking study by McIntyre et al. [9] revealed similar patterns, with most non-alignment methods having either low precision and high recall or vice versa. This makes sense intuitively as it is easy to select only a few high-confidence species as being present or to claim that almost all known species are present; conversely, balancing precision and recall presents a substantial challenge. This presents a difficult trade-off to researchers, who must choose between failing to identify most organisms and falsely identifying many organisms. Methods that ranked among the worst in recall and abundance estimation in the CAMI competition [7] and other benchmarking studies [9, 10] have been used in large-scale efforts to analyze human [11] and city metro [12] microbiomes and have also been used to perform downstream analyses that link the microbiome to host genetics [13] and diseases such as colorectal cancer, potentially impacting the findings of these important studies.

We developed Metalign to address common obstacles to metagenomic analyses. Metalign is an efficient alignment-based metagenomic profiling method that achieves a strong balance of precision and recall with runtimes comparable to the state-of-the-art methods. Alignment-based profiling is regarded as highly accurate, but aligning millions of reads against a reference database of tens to hundreds of gigabytes (GB) in size is computationally infeasible. Metalign minimizes computational cost with a high-speed, high-recall pre-filtering method based on the mathematical concept of containment min hash [14], which identifies a small number of candidate organisms that are potentially in the sample and creates a subset database consisting of these organisms. This pre-filtering approach reduced our comprehensive NCBI-based database of 243 GB, often by more than 100-fold, with some variance depending on the diversity of the sample. We limit false positives by performing a highly accurate alignment step on the subset database, which handles both the reads that align uniquely to one genome and the reads that align to multiple genomes. Metalign then profiles the organisms that reach a certain threshold amount of reads uniquely aligned to their genome, along with other optional metrics (see the “Methods” section).

We compared the performance of Metalign and several state-of-the-art profiling methods on both simulated data from the CAMI competition [7] and real data from an in vitro mock community [15] and the Tara Oceans project [16]. We found that Metalign substantially outperforms all other methods on one or both datasets, with the exception of one method that performs similarly to Metalign but with substantially slower running time. Metalign should benefit researchers seeking to efficiently obtain highly accurate metagenomic community profiles, enabling more accurate scientific discovery and downstream analysis.

## Results

### Methods overview

**Fig. 1:**
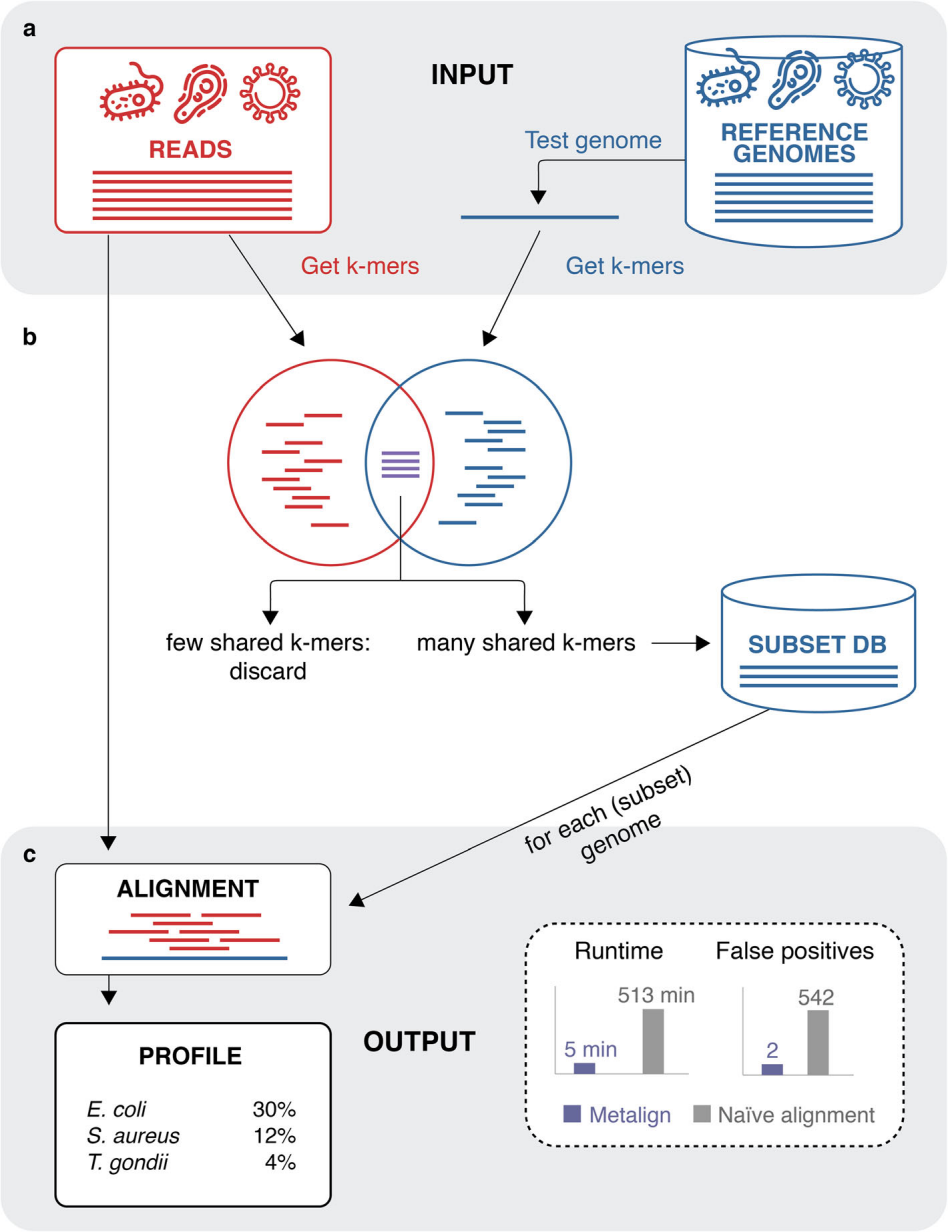
Metalign overview. **a** The input to Metalign is sequencing reads and a reference database. **b** The pre-filtering stage, based on an implementation of the theoretical concept of containment min hash [14], quickly estimates the percentage of *k*-mers in each reference genome that are also in the reads. Metalign then selects a small “subset” database consisting of reference genomes above a certain containment percentage threshold. **c** Metalign then performs alignment between the reads and the reference genomes in the subset database, outputting a profile in the standardized, community-driven format used by OPAL [17] and CAMI [7]. Applying Metalign to in vitro mock community data compared with naive alignment without pre-filtering reduced runtime from 513 to 5 min and false-positive genera from 542 to 2

Aligning millions of reads to reference databases—which are hundreds of gigabytes in size—is computationally infeasible. However, with an effective pre-filtering stage (Fig. 1a), highly accurate alignment can be performed on the small pre-filtered database.

We use KMC [18] to generate *k*-mers for the reads and each reference genome, and then, we utilize an implementation of the theoretical concept of containment min hash [14] (called CMash) to estimate the percent of *k*-mers in each reference genome that are also present in the reads (the “Methods” section). Intuitively, this gives us an estimate of how likely it is for each reference genome to be present in the sample. We place in a subset database only the reference genomes above a certain percentage cutoff threshold, to which we align the reads using the fast modern aligner Minimap2 [19]. Finally, we estimate the relative abundances of microbes in the sample by combining information from reads that map uniquely to one genome with those that align to multiple genomes (see the “Methods” section).

We tested the efficacy of our CMash database pre-filtering step (the “Methods” section) by running alignment with Minimap2 on our full unfiltered database and on the reduced database produced by our pre-filtering step. We then applied the alignment and profiling stage of Metalign. We performed these experiments on a mock community dataset consisting of 300,969 reads, obtained from Peabody et al. [15]. Alignment and profiling with the full database took approximately 513 min, while alignment and profiling with the CMash-reduced database—enabled by Metalign—took approximately 5 min. Additionally, the pre-filtering step reduced noise in the form of spurious alignments to organisms that were not present in the data. Consequently, while both strategies detected all genera present in the data, profiling using the full unfiltered database produced 542 false-positive genera, while profiling using the pre-filtered database produced only two false positives. This experiment highlights the significant improvements in speed and precision enabled by Metalign’s CMash-based pre-filtering step.

### Metalign achieves state-of-the-art performance on CAMI simulated data

The Critical Assessment of Metagenome Interpretation (CAMI) [7] provides the most comprehensive and in-depth evaluation of metagenomic profiling, binning, and assembly methods to date. In the profiling competition, many of the most well-known methods were evaluated on a variety of simulated datasets that modeled real-life challenges, such as various community diversities and confounding sequences from high-abundance plasmids and novel viral strains. We evaluated the performance of the top-ranked methods in terms of several metrics: recall, precision, F1 score, Jaccard index, L1 norm error, and weighted UniFrac (Supplementary Information). In total, there were eight datasets: one low-diversity community, two medium-diversity communities, and five high-diversity communities. Each dataset consisted of 15 Gbp of sequence data (further details on these communities are available in the CAMI paper [7]). Of note, the high-complexity communities contained many organisms not represented in the Metalign training database. For example, in one of the CAMI high-complexity datasets, Metalign’s database contained only 161 out of the 243 unique species in the dataset. Additionally, a high percentage of the total sample abundance is composed of unmappable reads; for example, in the aforementioned dataset, over 71.4% of the sample abundance consists of novel strains.

We ran Metalign on these eight datasets and compared our results with several other state-of-the-art methods: Kraken2 [20] with its abundance estimation companion method Bracken [21] (“Bracken+Kraken2”), CLARK [9], MetaPhlAn2 [22], mOTUs2 [23], MetaBinG2 [24], GOTTCHA [25], and MEGAN6 [26, 27] with its associated fast alignment method DIAMOND [28] (“MEGAN+DIAMOND”). The first four of these methods were chosen for their wide usage and past involvement with the CAMI competition, and the latter two were added because of their strong performance on benchmarking studies by McIntyre et al. [9] and Ye et al. [10].

**Fig. 2:**
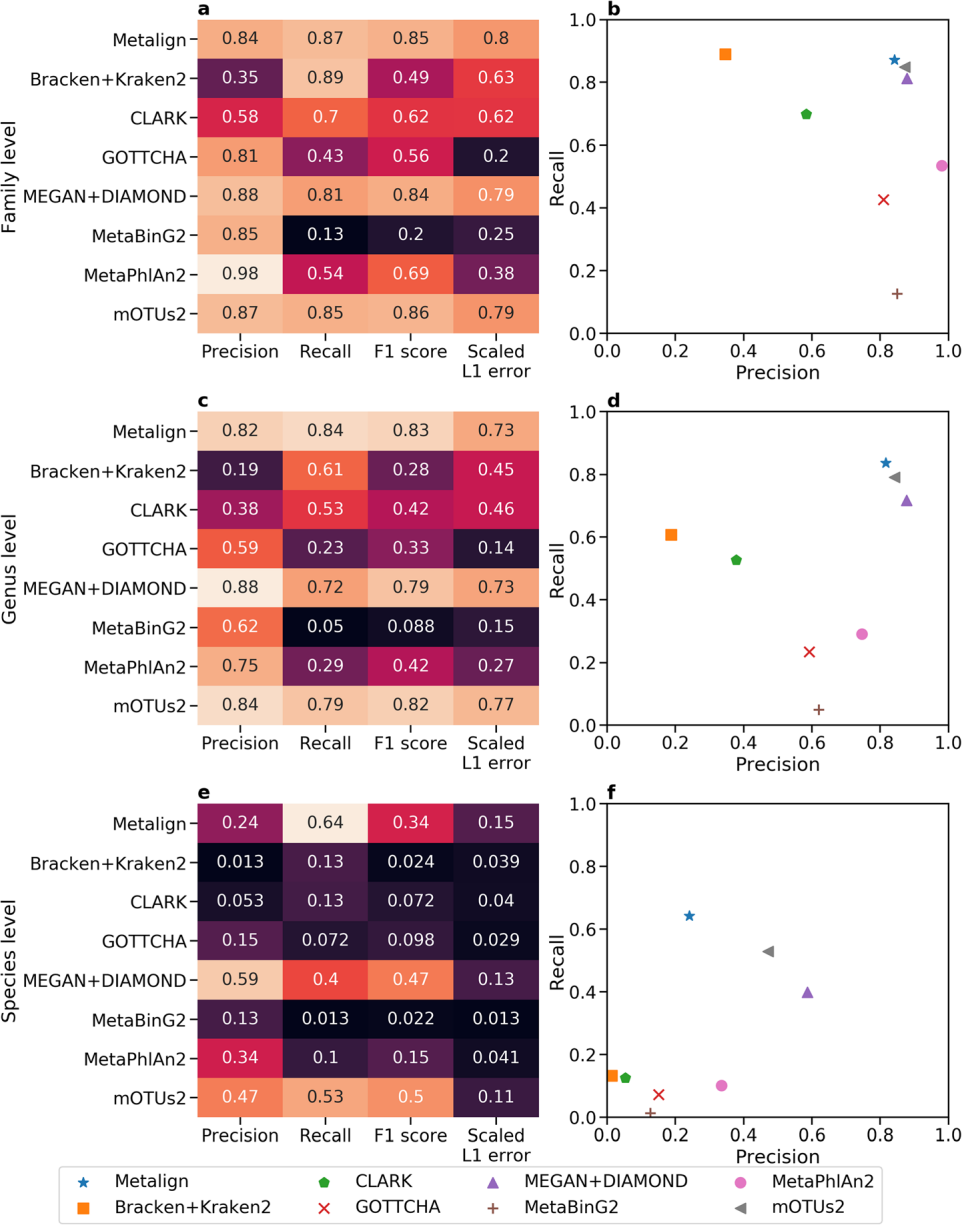
Comparison of Metalign with competing methods on the challenge datasets from the first CAMI competition with organisms below 0.01% relative abundance excluded. Heatmaps show precision, recall, F1 score, and L1 error re-scaled such that higher is better (1 − (L1 error/2)) at the **a** family level, **c** genus level, and **e** species level. Scatter plots show Precision (*x*-axis) versus recall (*y*-axis) at the **b** family level, **d** genus level, and **f** species level. Metrics were averaged across all eight datasets

**Fig. 3:**
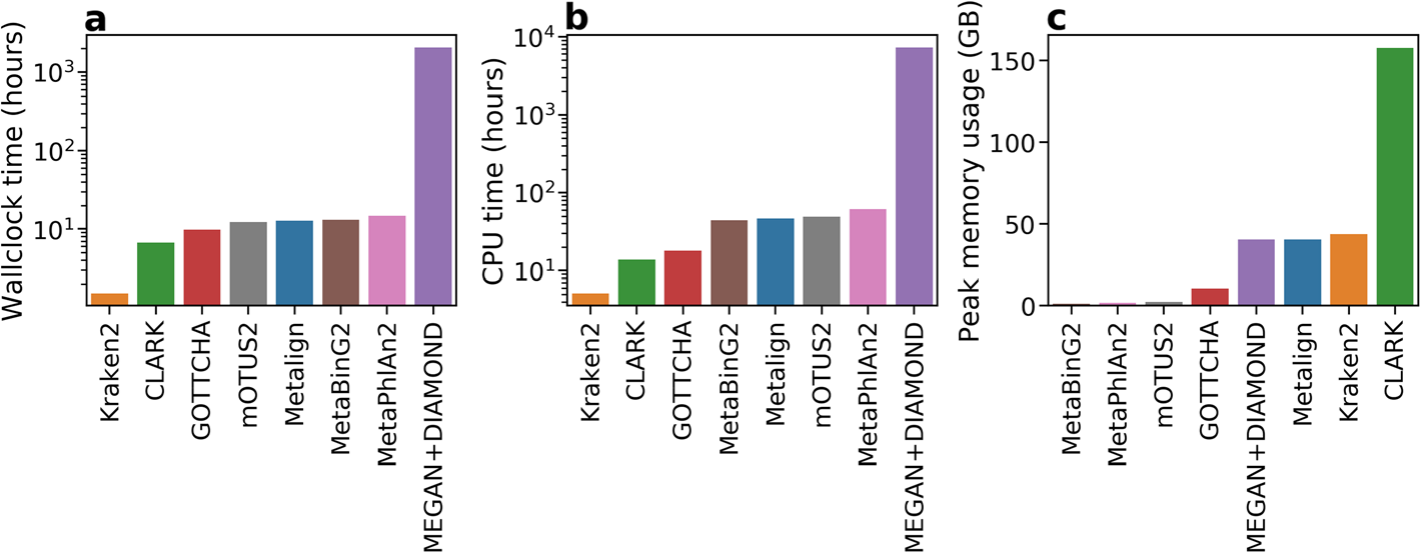
Comparison of Metalign with competing methods on the challenge datasets from the first CAMI competition in **a** wall clock running time, **b** CPU time, and **c** peak memory usage in gigabytes. The running times were summed across all eight datasets, while the memory values reflect the peak memory usage across all eight datasets. All methods were run with 4 threads

The comparisons were performed using the CAMI-affiliated evaluation software OPAL [17] (Fig. 2), and timing and memory usage statistics were collected (Fig. 3). Each method was run with its default database; CLARK was set to detect organisms from all available taxonomic ranks. Because methods such as CLARK and Kraken2 are known to produce a large number of low-abundance false positives [7, 9, 15], we removed organisms assigned less than 0.01% abundance from the profiles of all methods. This cutoff threshold resulted in a better F1 score for these methods than not using a threshold (Additional file 1: Fig. S1). Further details on how the tools were run are available in the Supplementary Information.

Metalign, MEGAN+DIAMOND, and mOTUs2 substantially outperformed Bracken+Kraken2, CLARK, GOTTCHA, MetaBinG2, and MetaPhlan2 in both presence/absence prediction (F1 score) and abundance estimation (L1 error). As expected from previous studies [7, 9, 15], Bracken+Kraken2 and CLARK produce results with high false-positive rates. However, regardless of the abundance cutoff threshold chosen, these methods produce an F1 score that falls short of the top-performing methods. MetaPhlan2 and GOTTCHA produce results with strong precision but comparatively high false-negative rate and abundance estimation error, as noted in previous studies [7, 9]. MetaBinG2, like GOTTCHA, had good precision but poor recall and abundance estimation. Metalign, MEGAN+DIAMOND, and mOTUs2 performed similarly on most performance metrics, except weighted UniFrac, where mOTUs2 outperformed all other methods. Metalign is the next-best method, and MEGAN+DIAMOND ranks sixth out of the eight methods assessed (Additional file 1: Fig. S2).

Metalign ranked near the middle of the methods in terms of runtime and memory. As demonstrated by previous benchmarking studies [9, 10], MEGAN+DIAMOND was much slower than all other methods (Fig. 3a, b), taking 2072.3 wall clock hours while no other methods took more than 15 h. Kraken2 was the fastest method (1.5 wall clock hours to run on all datasets), followed by CLARK (6.7 h), with all other methods taking between 9 and 15 wall clock hours (Fig. 3a, b). Meanwhile, CLARK had very high memory requirements (157.6 GB peak memory usage), while MetaBinG2, MetaPhlAn2, and mOTUs2 had very low memory requirements (less than 2.5 GB). GOTTCHA had a peak memory usage of 10.5 GB; Metalign, Kraken2, and MEGAN+DIAMOND used 40–44 GB (Fig. 3c).

We repeated this experiment with Bracken+Kraken2 using the same custom database as Metalign with no abundance cutoff (Additional file 1: Fig. S3) and with a 0.01% abundance cutoff (Additional file 1: Fig. S4). Using Metalign’s custom database, Kraken2 achieved much better precision than with its original database, but Kraken2’s precision and F1 score were still lower across all taxonomic ranks evaluated and both abundance cutoff settings when compared to Metalign. For example, Kraken2’s precision at the genus level using a 0.01% abundance cutoff increased to 63.5% compared with 18.7% using its original database but was still lower than Metalign’s 81.7% precision; similarly, its F1 score increased from 27.6 to 73.5% compared with Metalign’s 82.5%. Importantly, in terms of memory usage (Additional file 1: Fig. S5), Kraken2 peaked at 325.23 GB with the custom database, suggesting that the use of such a comprehensive database would be infeasible for many users. In comparison, Metalign’s peak memory usage was 40.37 GB.

Finally, we assessed whether the discrepancy in runtime between MEGAN+DIAMOND and other methods could be reduced by increasing the number of threads used (Additional file 1: Fig. S6). We ran Metalign and MEGAN+DIAMOND on the first 5 million reads of the low-complexity dataset from the first CAMI challenge with 4, 8, 16, and 32 threads. Both methods experienced a nearly linear speedup when using 8 threads instead of 4, but had diminishing improvements as the number of threads was scaled up to 16 and then 32. In all cases, Metalign was 20–50× faster than MEGAN+DIAMOND.

### Metalign outperforms existing methods across a wide range of cutoff settings on in vitro mock community data

Simulated data often fails to fully capture the noise present in environmental microbial communities. However, a ground truth is not available for the latter, making the calibration and comparative evaluation of competing methods difficult [29]. In vitro mock communities are communities of microbes specifically designed and cultured in the laboratory. Such mock communities offer the benefits of “real” data—albeit not from a natural environment—but with the advantage of an available ground truth. We selected for our comparison of the performance of the eight methods a dataset that consists of 11 bacterial species and is available from a metagenomics benchmarking study performed by Peabody et al. [15]. We only evaluated the presence/absence prediction, as precise ground truth relative abundance information is not available for this dataset.

**Fig. 4:**
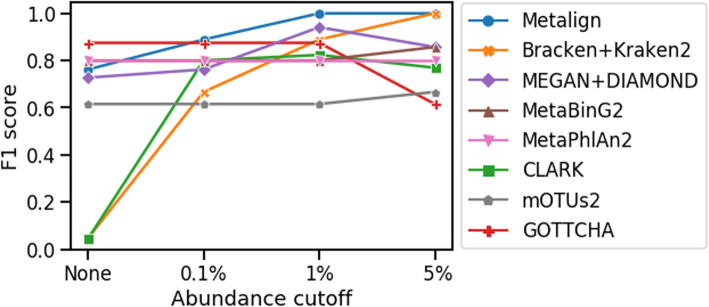
Genus-level F1 score at different abundance cutoff thresholds using in vitro mock community from Peabody et al. [15]. For a given cutoff threshold, we calculated the F1 score while leaving out predicted genera below the threshold

Previous studies have demonstrated that Kraken and CLARK are prone to generating a number of low abundance false positives [7, 9, 15]; therefore, it is common to employ an abundance cutoff threshold with these methods [15]. We evaluated the genus-level F1 score for these methods at a variety of cutoff thresholds (Fig. 4).

Metalign achieves the highest or tied-for-highest F1 scores among the tested methods at all cutoff thresholds, except in the case where no cutoff was used. Examining Metalign’s results, all eight true-positive genera were present with > 5% abundance, while a few false-positive genera with < 0.6% abundance were predicted, thus forming a clear separation between true and false positives. Metalign thus achieves a perfect F1 score with an abundance cutoff threshold of 1% or 5%.

Bracken+Kraken2 also achieves a 100% F1 score at a cutoff threshold of 5% but underperformed Metalign at other cutoff thresholds and had less of a clear separation between the lowest abundance true positive and the highest abundance false positive. Meanwhile, MetaBinG2, MetaPhlAn2, GOTTCHA, and MEGAN+DIAMOND performed fairly well across all cutoff settings, and CLARK performed fairly well when any cutoff threshold was applied. Unlike the experiments with the CAMI datasets, mOTUs2 performed poorly on the mock community data. Indeed, mOTUs2 was the worst method by a significant amount with the 0.1% and 1% cutoff thresholds and the second-worst method at the 5% threshold. mOTUs2 has previously been shown to perform well on the CAMI data [23] but less well on other datasets [10], and our results suggest a similar inconsistency.

**Fig. 5:**
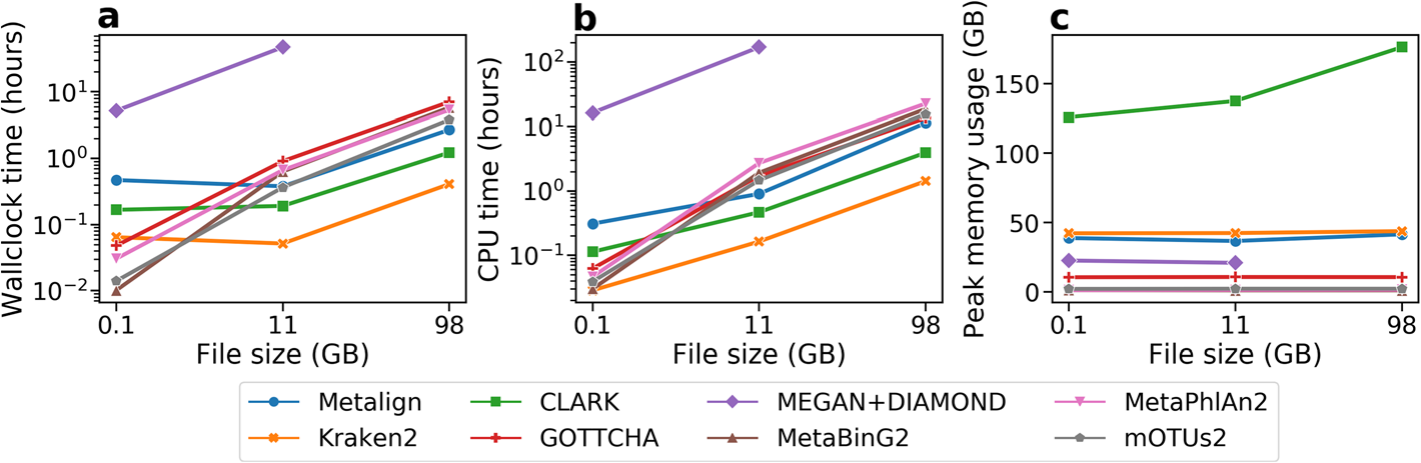
**a** Wall clock running time in hours, **b** CPU time in hours, and **c** peak memory usage in gigabytes of the tested methods on three real datasets of various sizes: the Peabody mock community (0.1 GB) and two samples from the Tara Oceans project (11 GB and 98 GB). All methods were run with 4 threads. In order to generate comparable files, the Peabody data was converted to FASTQ format and the separate paired-end and gzipped Tara Oceans files were decompressed and interleaved. MEGAN+DIAMOND did not finish running after 168 wall clock hours (1 week) on the 98-GB file

We also used real data to benchmark the methods in terms of wall clock runtime, CPU time, and peak memory usage on more realistic data (Fig. 5). We used two datasets from the Tara Oceans project [16] in order to evaluate the scaling performance of the methods from the small ~ 0.1 GB Peabody dataset to 11 GB and 98 GB datasets from the Tara Oceans project [16]. The Tara Oceans reads were originally in separate gzipped paired-end files, which we decompressed and interleaved with BBMap [30] before running the methods on this data.

Similarly to their performance when applied to simulated data, Kraken2 and CLARK were the fastest methods for both wall clock and CPU time when run on the Tara Oceans datasets, and MEGAN+DIAMOND was by far the slowest. The other methods had similar runtimes, with Metalign being slightly faster than MetaBinG2, MetaPhlAn2, mOTUs2, and GOTTCHA on the large datasets. For the 98-GB dataset, DIAMOND failed to finish within 1 week of run time, so this data point is absent. All other methods finished running on the 98-GB dataset in less than 8 h.

Interestingly, while the running times of MetaBinG2, MetaPhlAn2, mOTUs2, and GOTTCHA scaled roughly linearly with the size of the dataset, Metalign, Kraken2, and CLARK ran the 11 -GB Tara Oceans dataset in about the same amount of time as the 0.1-GB Peabody mock community dataset. This is likely because far fewer reads (or *k*-mers) could be mapped/assigned from the Tara Oceans dataset, which contains many unknown marine organisms, compared with the Peabody dataset which consists of a small number of well-known organisms. In addition, these methods all have database loading times that do not scale with the dataset size. This explains their slower running time on the Peabody dataset.

In terms of memory usage, CLARK remained the highest by far while MetaPhlAn2, mOTUs2, and MetaBinG2 were the lowest, and the rest of the methods were in-between. Most methods used roughly the same amount of memory regardless of file size, except CLARK, whose memory usage increased sub-linearly with the size of the input file.

### Metalign advantages compared to top-performing methods

We compared the performance of tools when applied to low-diversity in vitro mock communities consisting of well-characterized organisms, and the high-diversity CAMI simulated data, which contained a large number of novel organisms. Our study shows that many tools struggled to maintain high precision, high recall, and accurate abundance estimation in both of these experiments. For example, mOTUs2 performed well on the CAMI-simulated data but was the worst-performing method on the mock community data (Fig. 4), while Bracken+Kraken2, CLARK, GOTTCHA, MetaPhlAn2, and MetaBinG2 performed relatively well on the mock community data (assuming a moderate abundance cutoff threshold) but poorly on the simulated data (Figs. 4 and 2). Methods that performed poorly on the simulated data fall into two categories: high recall but low precision (Bracken+Kraken2) and high precision but low recall and high abundance estimation error (GOTTCHA, MetaPhlAn2, MetaBinG2).

In contrast, the alignment-based methods of Metalign and MEGAN+DIAMOND were the only two tools that maintained high precision and recall on both kinds of data while maintaining reasonable abundance estimation. However, MEGAN+DIAMOND required roughly two orders of magnitude more runtime when compared to all other methods (Figs. 3 and 5), becoming nearly infeasible on the CAMI datasets, each of which had only ~ 50 million paired-end reads. Indeed, on the largest Tara Oceans dataset (206.5 million paired-end reads), we were unable to run MEGAN+DIAMOND within 168 h of wall clock time (1 week) using four threads.

We tested whether or not the increased size of the training database of Metalign resulted in its superior performance to non-alignment-based techniques. We selected Bracken+Kraken2, as it is one of the few alignment-free methods that allows for custom training databases, and re-trained it on the same database as that used by Metalign (as mentioned in the previous section). This did significantly improve Bracken+Kraken2’s performance on the simulated data, particularly in precision (Figures S3 and S4), although Metalign still achieved a higher precision and F1 score.

However, Kraken2’s memory usage spiked to over 325 GB (Additional file 1: Fig. S5), making the use of this large training database with Kraken2 infeasible for many users with limited hardware.

As such, we find that Metalign is the only method we tested that scales well for both large samples and large reference databases, while maintaining a balance between high precision and high recall across datasets of differing diversity.

## Discussion

Metalign’s pre-filtering technique will continue to enable alignment-based metagenomic analysis techniques even as reference databases continue to grow in size. Using this approach, alignment is only performed on the portions of the reference database that are relevant to the input metagenomic sample. Given the speed and accuracy results, the resulting alignments of Metalign could be used for other analyses (e.g., microbial genome rearrangement, microbial genome-wide association studies) that would not be practically possible with a standard sequence aligner on the full database (as demonstrated by the run-time results depicted for DIAMOND in Fig. 5 and BLAST in Fig. 1). Additionally, Metalign’s database can be updated and re-trained in a straightforward manner, in contrast to marker gene-based methods such as MetaPhlAn2 and the alignment-based mOTUs2 [10].

Several directions hold promise for further improvements to Metalign. The default containment index cutoff for inclusion of organisms in the reduced database is currently set in an empirical and somewhat arbitrary fashion, but preliminary theoretical results indicate that the containment index is related to the well-known average nucleotide identity, which facilitates less arbitrary cutoff. The resolution of multi-aligned reads could be performed in a more robust manner at the expense of additional computational time, for example, by using base-level metrics such as quality scores and CIGAR strings [31]. The standard of evidence used by Metalign to determine the presence of an organism could incorporate genome coverage information or be automatically modulated based on the characteristics of the sample such as sequencing depth and estimated alpha diversity. Additionally, an iterative, Bayesian-like approach could leverage an initial taxonomic profile produced by Metalign as a prior upon subsequent re-assignment of multi-mapped reads. Similar Bayesian-like approaches are useful for related metagenomic analysis tasks [32].

Finally, several additional analyses could be employed to evaluate Metalign’s performance more comprehensively, including comparisons with more methods and performance comparisons on other benchmark datasets such as those released by McIntyre et al. [9] and Ye et al. [10]. All reference-based profiling methods will degrade in performance as the portion of organisms present in the sample but not in the reference database increases. This has been demonstrated empirically by Qiao et al. [24] using “clade exclusion experiments” to measure the relative performance degradation of different methods when taxonomic clades are excluded from the reference database. As Qiao et al. showed that alignment-based methods can struggle in these settings relative to composition-based methods like MetaBinG2 [24], it would be interesting to measure how much Metalign’s performance degrades in such experiments relative to other methods.

## Conclusions

We developed Metalign, a novel computational approach capable of achieving a strong balance between precision and recall across a variety of datasets, community diversities, and taxonomic levels. Several factors drive Metalign’s high performance. Metalign’s novel pre-filtering, database reduction step substantially quickens and increases the precision of downstream alignment and taxonomic profile inference due to the removal of organisms that cannot reasonably be contained in the sample (due to low containment index) but would instead just confound sequence alignment efforts. Metalign is able to obtain high precision because it requires an organism to pass the pre-filtering threshold and have at least one high-quality read mapping uniquely to its genome in the subsequent alignment stage. At the same time, Metalign maintains high recall by using a relatively liberal pre-filtering threshold that only excludes organisms which cannot reasonably be present and by not imposing excessive read alignment or genome coverage requirements in the mapping stage. Abundance estimation is improved by considering multi-aligned reads. For these reasons, we anticipate that Metalign will be useful to people seeking to generate accurate abundance estimation profiles from metagenomic sequencing data.

## Methods

### Database construction

In order to compile as comprehensive a reference database as possible, we used all NCBI microbial genome assemblies, including complete and incomplete assemblies from both RefSeq and GenBank. The final database, as of the writing of this paper, consisted of 199,807 organisms totaling 243 GB in size when gzipped. We intend to continue updating the reference database alongside NCBI database updates. We use rsync to download all NCBI genomes and then filter out unwanted taxa such as animals and plants. The script for this procedure is available on our GitHub repository (https://github.com/nlapier2/Metalign).

### Database pre-filtering with CMash

Aligning millions of reads to reference databases of hundreds of gigabytes in size is computationally infeasible. However, we show in this paper that aligning to a much smaller pre-filtered database can be done in a reasonable amount of time. Our pre-filtering stage uses a *k*-mer-based approach to focus on high speed and high recall (i.e., low false-negative rate). First, KMC [18] is used to enumerate the *k*-mers in the reads, with the *k*-mers of the reference genomes having been pre-computed by KMC, and then intersect these sets. We then utilize the containment MinHash similarity metric (presented theoretically in [14] via an implementation by one of the coauthors (Koslicki) called CMash, accessible via https://anaconda.org/bioconda/cmash or https://github.com/dkoslicki/CMash, to efficiently estimate the similarity/containment index) between each reference genome and the input sample. The containment index is closely related to the Jaccard index and, in this case, refers to the percent of *k*-mers in a reference genome that are also present in the reads.

Given the estimates for the containment index for each reference genome, we select all reference genomes above a certain cutoff threshold for inclusion in a new, reduced database on which to perform alignment. We currently set the cutoff to 0.01, as this value empirically gave us a good balance between recall and precision in our downstream results. Minor adjustments to this value generally have minor impacts on the results; however, setting the threshold too high produces false negatives while setting it too low results in low-abundance false positives.

We allow users to modulate the cutoff threshold, which it controls the balance between precision and recall, to suit their needs. By default, we keep only one strain per species (the strain with the highest containment index) above the cutoff threshold, and we discard extra strains. Metalign is capable of strain-level profiling via an optional software flag; however, in practice, profiling strains tends to slow down computation. Based on these user-set options, we construct a reduced database on which to perform alignment and profiling.

### Alignment and profile generation

Alignment of the reads to the pre-filtered database is performed using Minimap2 [19], which we empirically observed to generate accurate results similar to those produced by older alignment methods—but in much less time. Two main challenges arise with generating a taxonomic profile given mapping results: handling multi-aligned reads (i.e., reads successfully aligned to multiple reference genomes) and determining the threshold of evidence required to consider an organism or taxa present. Choices addressing these challenges may affect trade-offs between abundance estimation, precision, and recall.

Utilizing multi-aligned reads is critical to producing abundance estimation. For example, many reads can map to all organisms in a genus but not to organisms in any other genus. Discarding this information could cause under-estimation of the organisms with many multi-mapped reads. In Metalign, multi-aligned reads are resolved according to the uniquely mapped abundances of the organisms that a read is aligned to. First, we calculate the abundance estimates using only the uniquely aligned reads, holding aside reads that align to multiple genomes. Next, for each read that aligns to multiple genomes, we assign its abundance to the genomes it aligns to proportionally to the number of reads that align uniquely to each of those genomes. As a concrete example, assume species X has 6000 uniquely aligned reads, and species Y has 2000. A read aligned to both species X and species Y thus has 75% of its bases assigned to species X, and 25% of its bases assigned to species Y. Note that this means that we do not perform explicit read classification/binning; rather, we only try to obtain the relative abundances of organisms, as accurately as possible. This is because reads that align equally well to multiple genomes cannot be classified, but they do indicate that some portion of the sample abundance should be shared among those genomes and not the others.

To ensure that poor alignments are not counted, and that multi-aligned reads have aligned roughly equally well to all of its potential source organisms, we only consider reads that align at least 95% of bases. We empirically found that this allows slightly more flexibility for errors than requiring perfect alignments, while still retaining high fidelity. As long as one such read aligned uniquely to a genome, we counted that organism as present, while all genomes with no uniquely aligned reads were discarded. We found this rule to balance precision and recall effective. We allow users to control how many reads must uniquely align to an organism and what percentage of their bases needs to align to count an organism as present, allowing users to modulate false-positive rate and false-negative rate to suit their needs.

## Supplementary information

**Additional file 1.** Supplementary text (including computing environments, information needed for replication, and performance metrics evaluated) and supplementary figures.

**Additional file 2.** Review history.

## Data Availability

Metalign’s source code is available under the MIT License on GitHub at https://github.com/nlapier2/Metalign [33], and a Bioconda package is available at https://anaconda.org/bioconda/metalign. We used Metalign version 0.12.5, which can be obtained either via Bioconda or the GitHub release (https://github.com/nlapier2/Metalign/releases/tag/v0.12.5), to generate all results in this paper. This version of the software is also available as a permanent digital object stored on Zenodo (10.5281/zenodo.3959470) [34]. We also established a separate GitHub repository with scripts for replicating the results in this paper (https://github.com/nlapier2/metalign_paper_replication) [35]. We have included all commands run to generate results, raw results files (including timing), and intermediate processed results in our GitHub repository, as well as a Jupyter notebook with the code to generate the figures. We exclusively used publicly available datasets in this paper. For the CAMI 1 results, we used the results and datasets from the GigaDB dataset here: 10.5524/100344. We used the challenge datasets, not the toy datasets. For the medium complexity datasets, we used the insert size of 270 reads. The Peabody mock community data was retrieved using the link in their paper [15]. The prokaryote-isolated Tara Oceans reads used in this study are available on EBI here: https://www.ebi.ac.uk/ena/data/view/PRJEB1787. The run accessions we used were ERR598952 and ERR598957. Extra details on each file (e.g., sampling location, depth, isolation method) can be found by clicking on the sample accession corresponding to each of those run accessions. Information on the locations of some of the Tara Oceans stations can be found here: http://ocean-microbiome.embl.de/companion.html.
