# Supplementary material for: Metalign: efficient alignment-based metagenomic profiling via containment min hash
Source: Genome Biol. 2020 Sep 10;21:242. doi: 10.1186/s13059-020-02159-0 (PMC7488264; doi:10.1186/s13059-020-02159-0)
Supplement: Supplementary file 1 — Additional file 1. Supplementary text (including computing environments, information needed for replication, and performance metrics evaluated) and supplementary figures. [file 13059_2020_2159_MOESM1_ESM.zip › Additional file 1.docx]

**Supplementary Information**

**Computing Environments**

All experiments, with a few exceptions discussed next, were run on a server running Ubuntu 16.04.3 LTS (Xenial Xerus) with an 80 core Intel(R) Xeon(R) CPU E5-2698 v4 processor operating at 2.2 GHz and with 528 GB of RAM. A small number of experiments had to be run in different computing environments due to resource limitations when using the aforementioned server. Due to issues with memory usage, CLARK and Kraken2 (when trained on Metalign’s database) were run on a server running Ubuntu 18.04.4 with four AMD EPYC 7742 64-Core processors operating at 3.3GHz and with 1TB of RAM. Due to issues with running time, MEGAN+DIAMOND was run on the CAMI 1 data on a computing cluster, using various Linux compute nodes with 64 GB of RAM.

**Replication**

We used Metalign version 0.12.5, which can be obtained either via Bioconda or the GitHub release (<https://github.com/nlapier2/Metalign/releases/tag/v0.12.5>), to generate all results in this paper. We also established a separate GitHub repository with scripts for replicating the results in this paper (<https://github.com/nlapier2/metalign_paper_replication>). We have included all commands run to generate results, raw results files (including timing), and intermediate processed results in our GitHub repository, as well as a Jupyter notebook with the code to generate the figures.

For the other methods, we used their default settings (except for the number of threads, which we set to 4 for timing purposes). For MetaPhlAn2, mOTUs2, and Bracken+Kraken2, we used their default databases as of October 2019 (except for the re-training of Bracken+Kraken2 discussed in the main text), while we used the default databases as of April 2020 for MEGAN+DIAMOND, GOTTCHA, and CLARK, and May 2020 for MetaBinG2. The exact commands run can be found in the repository above.

Metalign and mOTUs2 output their results in CAMI/OPAL format by default, while we had to convert the output of other methods to that format for the purpose of comparing performance. Conversion was straightforward for MEGAN+DIAMOND, Bracken, and CLARK, because they provide NCBI TaxIDs, which can then be used to reconstruct the proper TaxID and organism name lineage information via NCBI’s names.dmp and nodes.dmp files, which can be obtained via the NCBI FTP site (ftp://[ftp.ncbi.nih.gov/pub/taxonomy/new_taxdump/new_taxdump.zip](http://ftp.ncbi.nih.gov/pub/taxonomy/new_taxdump/new_taxdump.zip)). However, conversion for GOTTCHA was more challenge because it does not provide the TaxIDs. We converted the provided organism names to TaxIDs, then repeated the previously described procedure. MetaBinG2 provides GI numbers, which we converted to TaxIDs and then repeated the previously described procedure. The scripts we used to do these format conversions are on the Metalign replication GitHub repository. MetaBinG2 requires that reads be provided in FASTA format, so we converted reads files to FASTA using BBMAP [(30)](https://paperpile.com/c/uvOtyj/blSPK) before running MetaBinG2.

Due to the large running time of DIAMOND on the largest datasets (the CAMI datasets and the 98 GB Tara Oceans dataset), these jobs timed out after two days on the server on which we ran our other experiments. We were able to run DIAMOND on the CAMI files by de-interleaving them into two separate paired files and then running them on a separate server where we had a one-week time limit. These jobs completed in about 4-6 days each and the results were returned to our main server for “meganizing” to produce files containing read counts for each TaxID. Then, the meganized files for the separate paired ends of each file were combined by summing the two read counts for each TaxID and then converted to CAMI/OPAL format. While not ideal, this allowed us to complete our evaluation of MEGAN+DIAMOND on this data, and the method achieved strong performance. Unfortunately, we were unable to run DIAMOND on the 98 GB Tara Oceans dataset within a weeklong time limit.

**Performance Metrics**

We evaluate the performance of multiple methods using several different metrics, which are designed to encompass both performance in the binary classification task of predicting species presence or absence, and in the estimation of relative abundances of taxa. For species presence and absence, a "True Positive" (TP) indicates that a species that is actually present in a sample is correctly predicted as being present by a method, while a "False Positive" (FP) indicates that the method predicted the presence of a species that is absent from a sample and a "False Negative" (FN) indicates that a species was actually present in a sample but a method did not predict its presence. We use two metrics to assess the performance of a method in species presence/absence, precision and recall, defined below. Additionally, we report the F1-Score, which is defined as the harmonic mean of precision and recall. All three of these metrics range from 0 to 1, or 0% to 100%. These metrics can be written as follows:

[
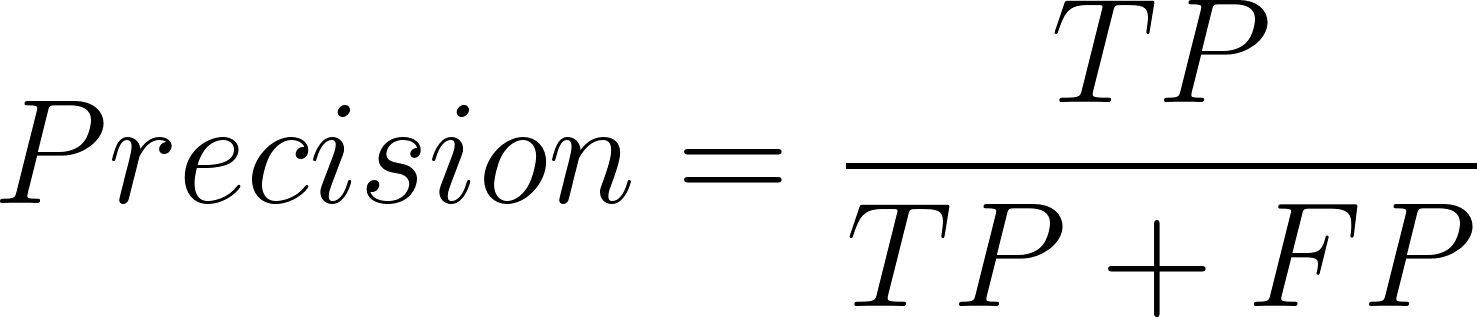
](https://www.codecogs.com/eqnedit.php?latex=Precision%20%3D%20%5Cfrac%7BTP%7D%7BTP%2BFP%7D#0)

[
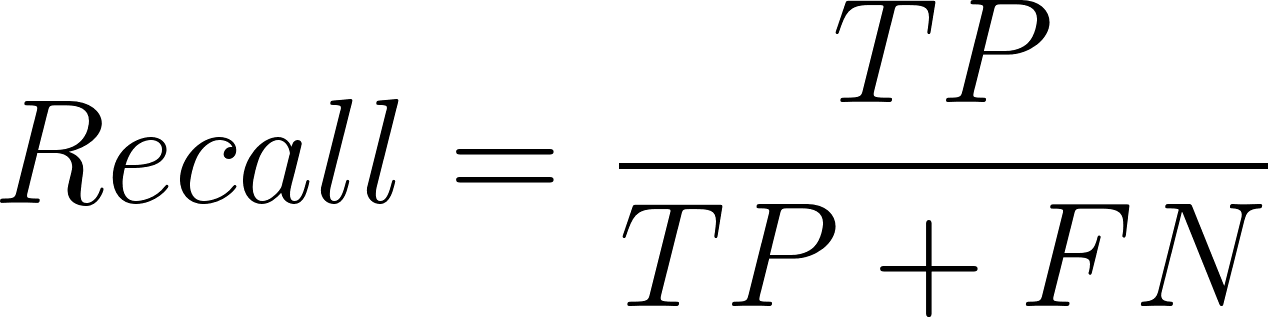
](https://www.codecogs.com/eqnedit.php?latex=Recall%20%3D%20%5Cfrac%7BTP%7D%7BTP%2BFN%7D#0)

[
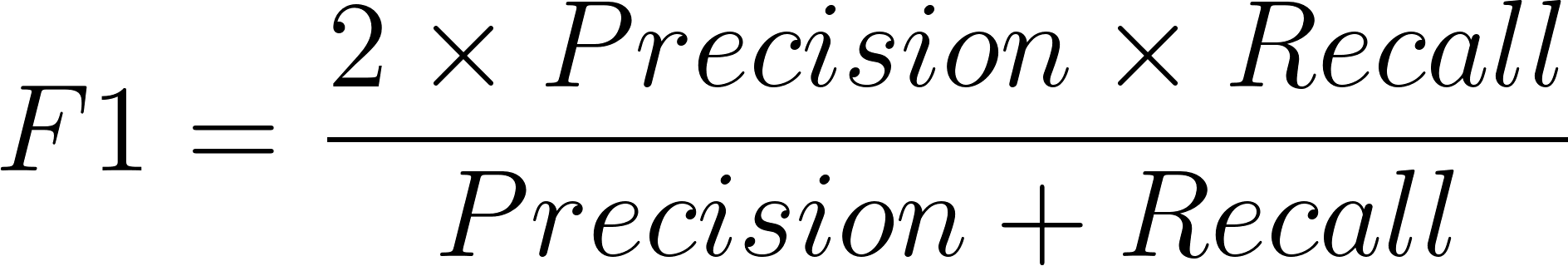
](https://www.codecogs.com/eqnedit.php?latex=F1%20%3D%20%5Cfrac%7B2%20%5Ctimes%20Precision%20%5Ctimes%20Recall%7D%7BPrecision%20%2B%20Recall%7D#0)

We use the L1-error as a measure of how accurately a method computes the relative abundances of species in a sample. The L1-error is the sum of absolute value differences between predicted species abundances and actual species abundances, and ranges from 0 (completely correct) to 2 (completely incorrect). An L1-error of 0 indicates that the exact set of actual species and their actual abundances is predicted perfectly, while a score of 2 indicates that the set of predicted species is completely incorrect. The L1-error can be described mathematically as:

[
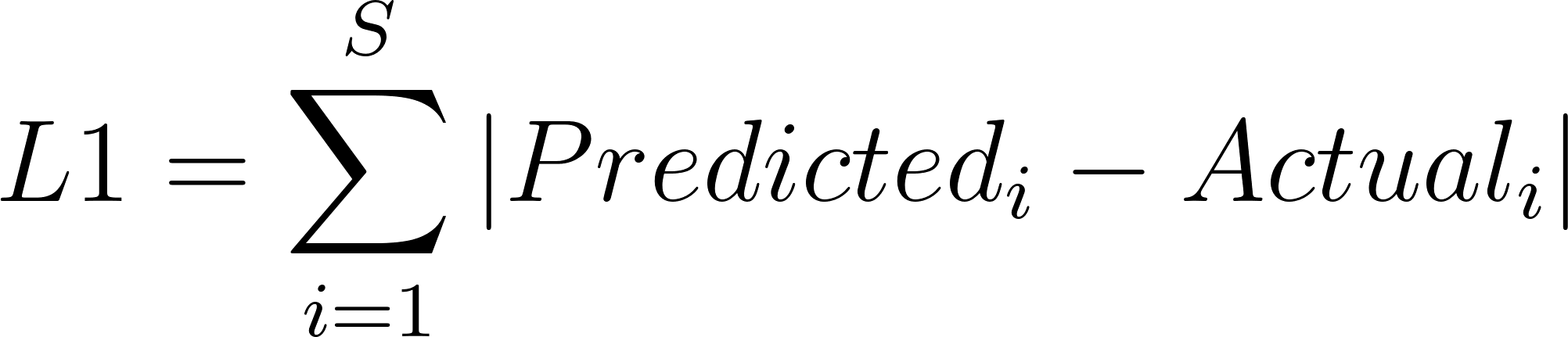
](https://www.codecogs.com/eqnedit.php?latex=L1%20%3D%20%5Csum_%7Bi%3D1%7D%5ES%20%7C%20Predicted_i%20-%20Actual_i%20%7C#0)

where S is the set of taxa that are predicted or actually present and i is the summation index. Finally, UniFrac [(36,37)](https://paperpile.com/c/uvOtyj/4PR9o+YTuXr) is a widely-used metric that measures differences between two microbial communities as the fraction of branch lengths of a phylogenetic tree that are present in one community or the other, but not both. More details on UniFrac and Weighted UniFrac can be found in the original papers by Lozupone et al. [(36,37)](https://paperpile.com/c/uvOtyj/YTuXr+4PR9o).

**Supplementary Figures**


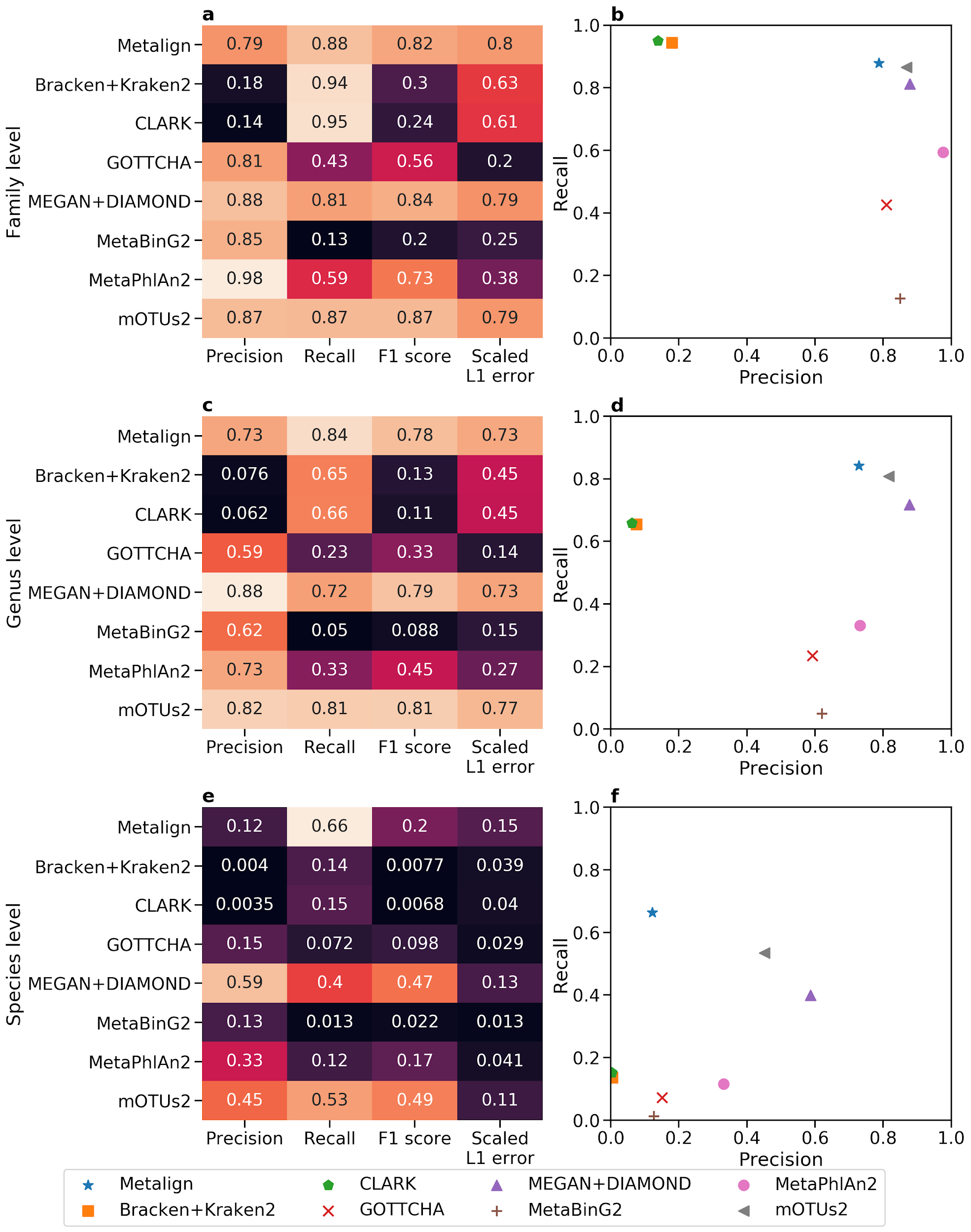


**Fig S1.** Comparison of Metalign and competing methods when applied to the challenge datasets from the first CAMI competition with no abundance threshold. Heatmaps show Precision, Recall, F1 score, and L1 error re-scaled such that higher is better (1 - (L1 error / 2)) at the (**a)** family level, (**c**) genus level, and (**e**) species level. Scatter plots show Precision (x axis) versus Recall (y axis) at the (**b)** family level, (**d**) genus level, and (**f**) species level. Metrics were averaged across all eight datasets.


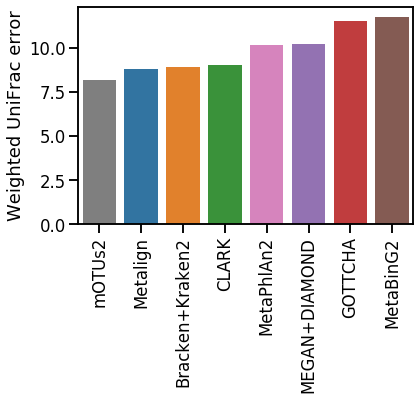


**Fig S2.** Comparison of Metalign and competing methods, with no abundance cutoff, when applied to the challenge datasets from the first CAMI competition on the Weighted UniFrac metric, which is rank-independent (lower is better).


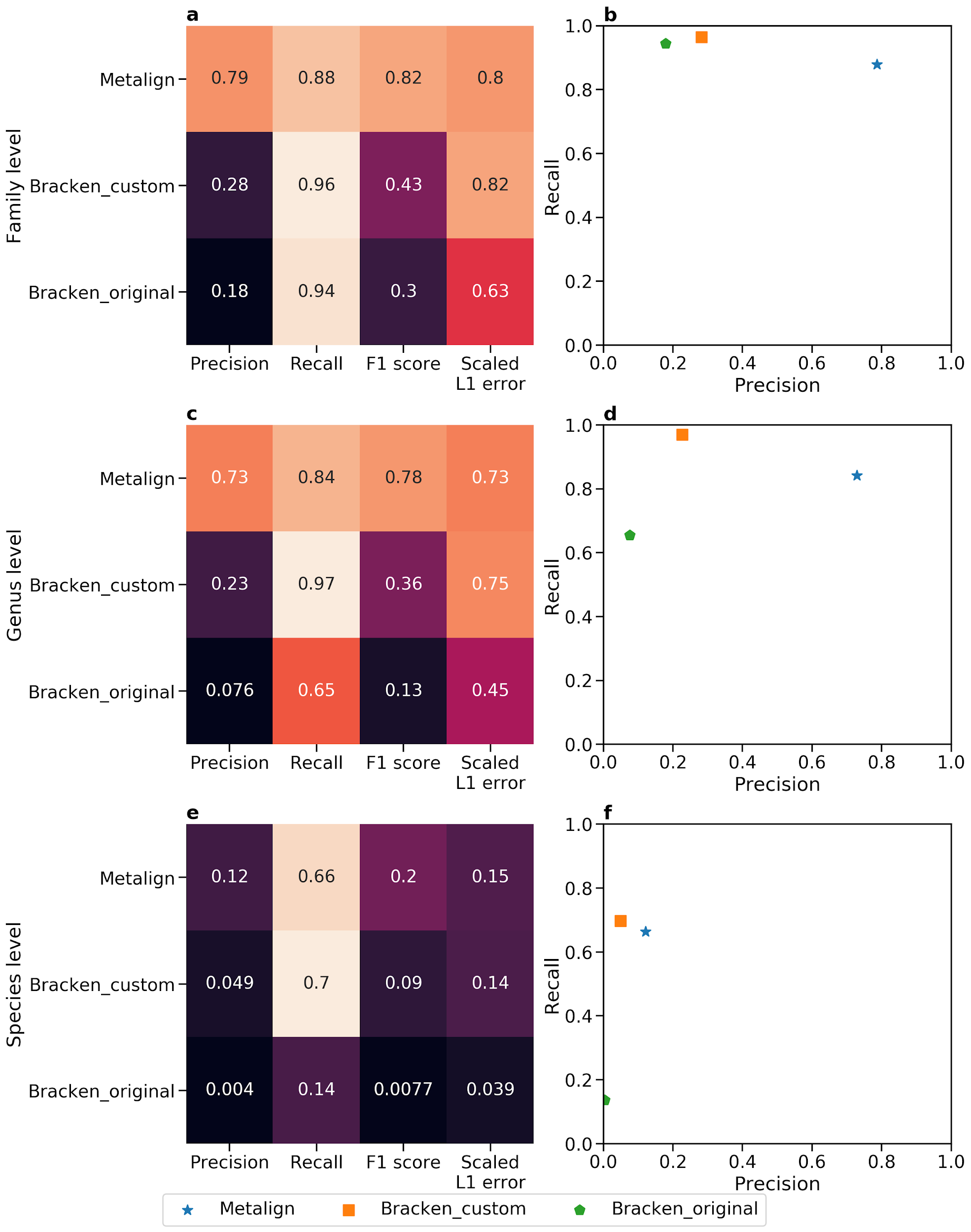


**Fig S3.** Comparison of Metalign Bracken+Kraken2 with the same custom database as Metalign, and Bracken+Kraken2 with its original database when applied to the challenge datasets from the first CAMI competition with no abundance threshold. Heatmaps show Precision, Recall, F1 score, and L1 error re-scaled such that higher is better (1 - (L1 error / 2)) at the (**a)** family level, (**c**) genus level, and (**e**) species level. Scatter plots show Precision (x axis) versus Recall (y axis) at the (**b)** family level, (**d**) genus level, and (**f**) species level. Metrics were averaged across all eight datasets.


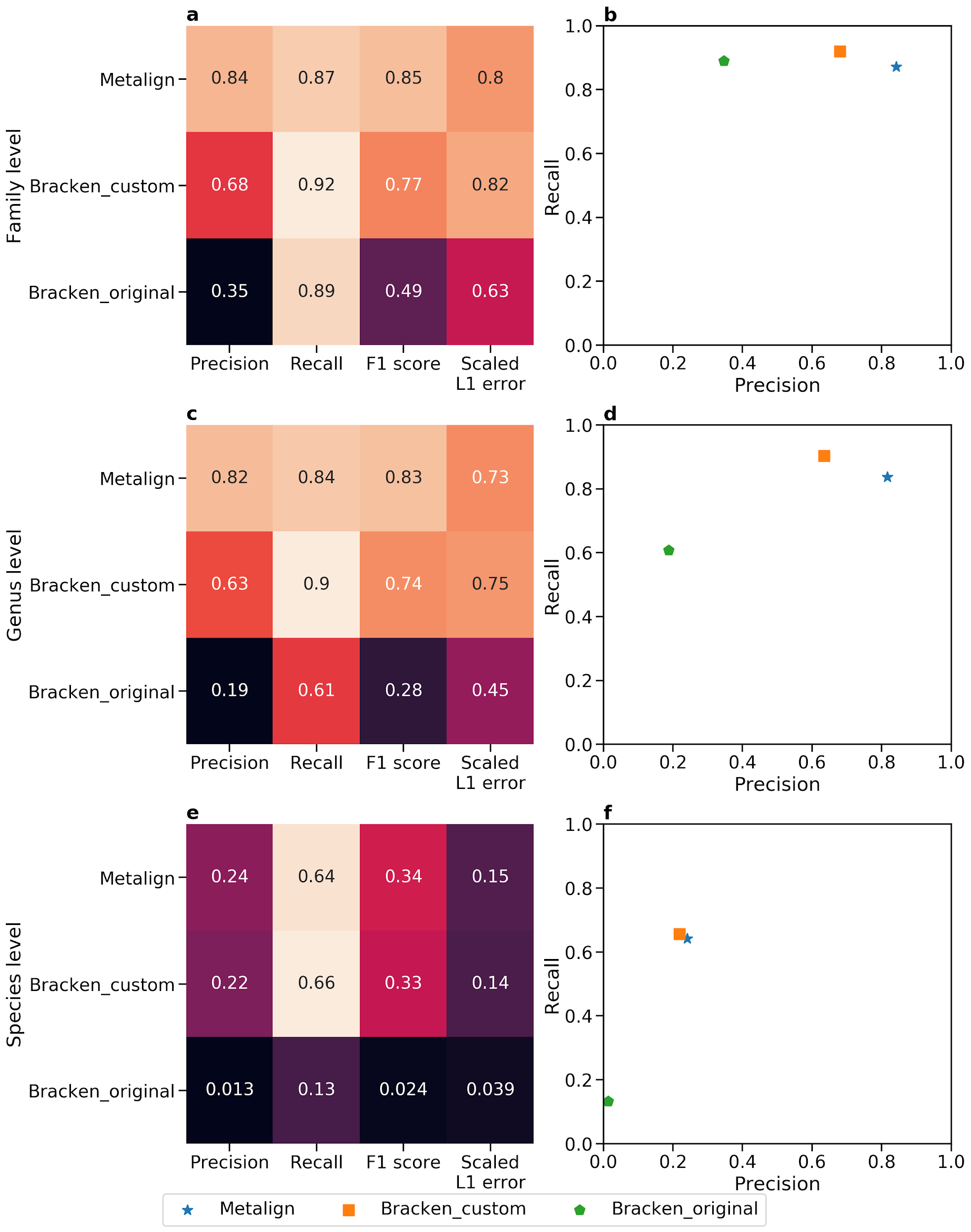


**Fig S4.** Comparison of Metalign, Bracken+Kraken2 with the same custom database as Metalign, and Bracken+Kraken2 with its original database when applied to the challenge datasets from the first CAMI competition with organisms below 0.01% relative abundance excluded. Heatmaps show Precision, Recall, F1 score, and L1 error re-scaled such that higher is better (1 - (L1 error / 2)) at the (**a)** family level, (**c**) genus level, and (**e**) species level. Scatter plots show Precision (x axis) versus Recall (y axis) at the (**b)** family level, (**d**) genus level, and (**f**) species level. Metrics were averaged across all eight datasets.


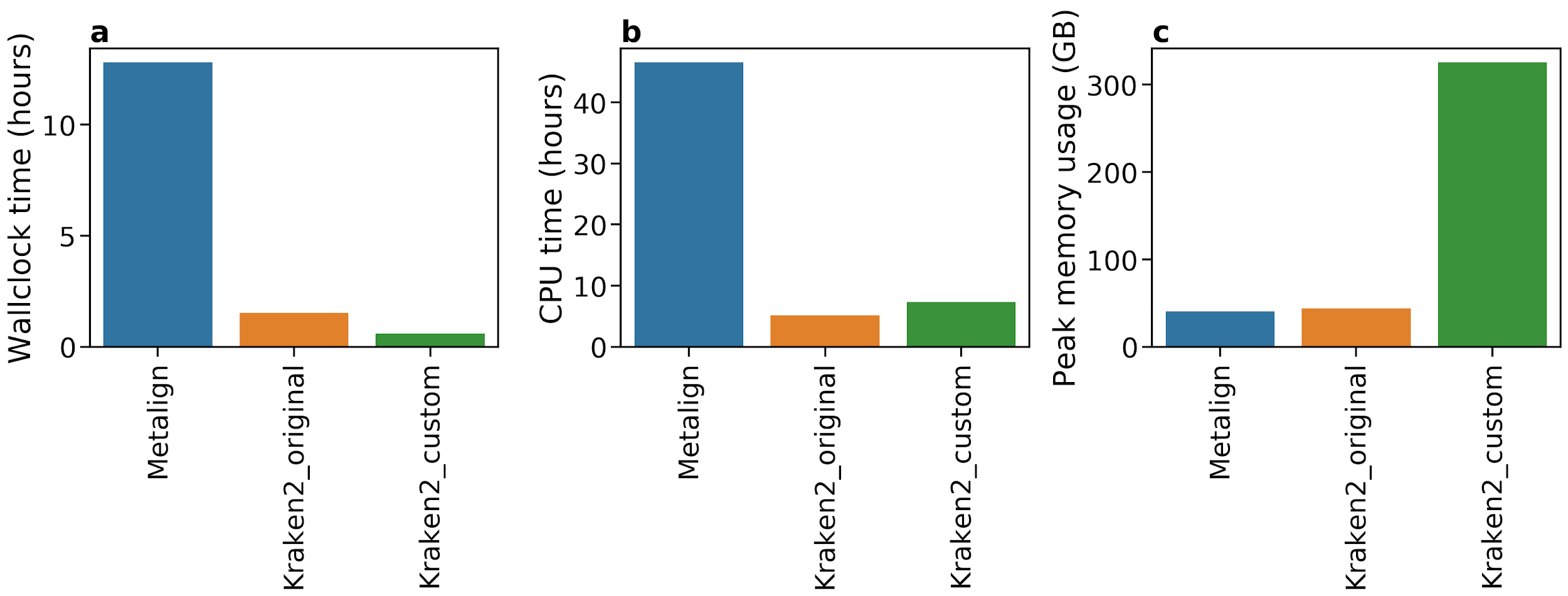


**Fig S5.** Comparison of Metalign, Kraken2 with the same custom database as Metalign, and Kraken2 with its original database when applied to the challenge datasets from the first CAMI competition in (**a**) wallclock running time, (**b**) CPU time, and (**c**) peak memory usage. The running times were summed across all eight datasets, while the memory values reflect the peak memory usage across all eight datasets. All methods were run with 4 threads. Due to computing resource limitations, Kraken2 with the custom database was run on a different server with more memory capacity and more compute threads, explaining its higher CPU time relative to wallclock time compared to its default database.


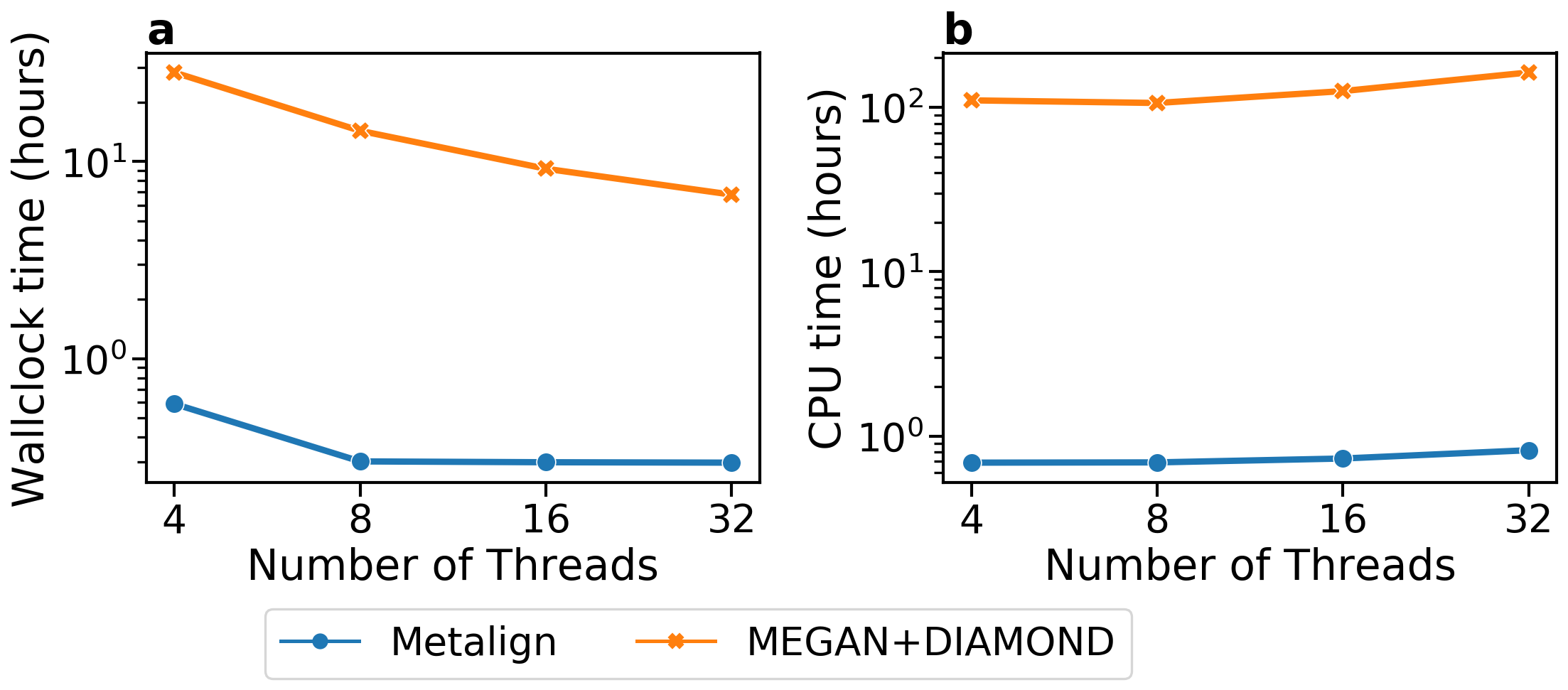


**Fig S6.** Comparison of Metalign and MEGAN+DIAMOND in terms of (**a**) wall clock time and (**b**) CPU time as they scale with the number of threads used (4, 8, 16, or 32). The dataset tested is the first 5 million reads of the low complexity dataset from the first CAMI challenge.

**References**

36. Lozupone CA, Hamady M, Kelley ST, Knight R. Quantitative and qualitative β diversity measures lead to different insights into factors that structure microbial communities. Appl Environ Microbiol. 2007;73(5):1576–85.

37. Lozupone C, Knight R. UniFrac: a new phylogenetic method for comparing microbial communities. Appl Environ Microbiol. 2005;71(12):8228–35.
