# Supplementary figures and images for: Metalign: efficient alignment-based metagenomic profiling via containment min hash
Source: Genome Biol. 2020 Sep 10;21:242. doi: 10.1186/s13059-020-02159-0 (PMC7488264; doi:10.1186/s13059-020-02159-0)

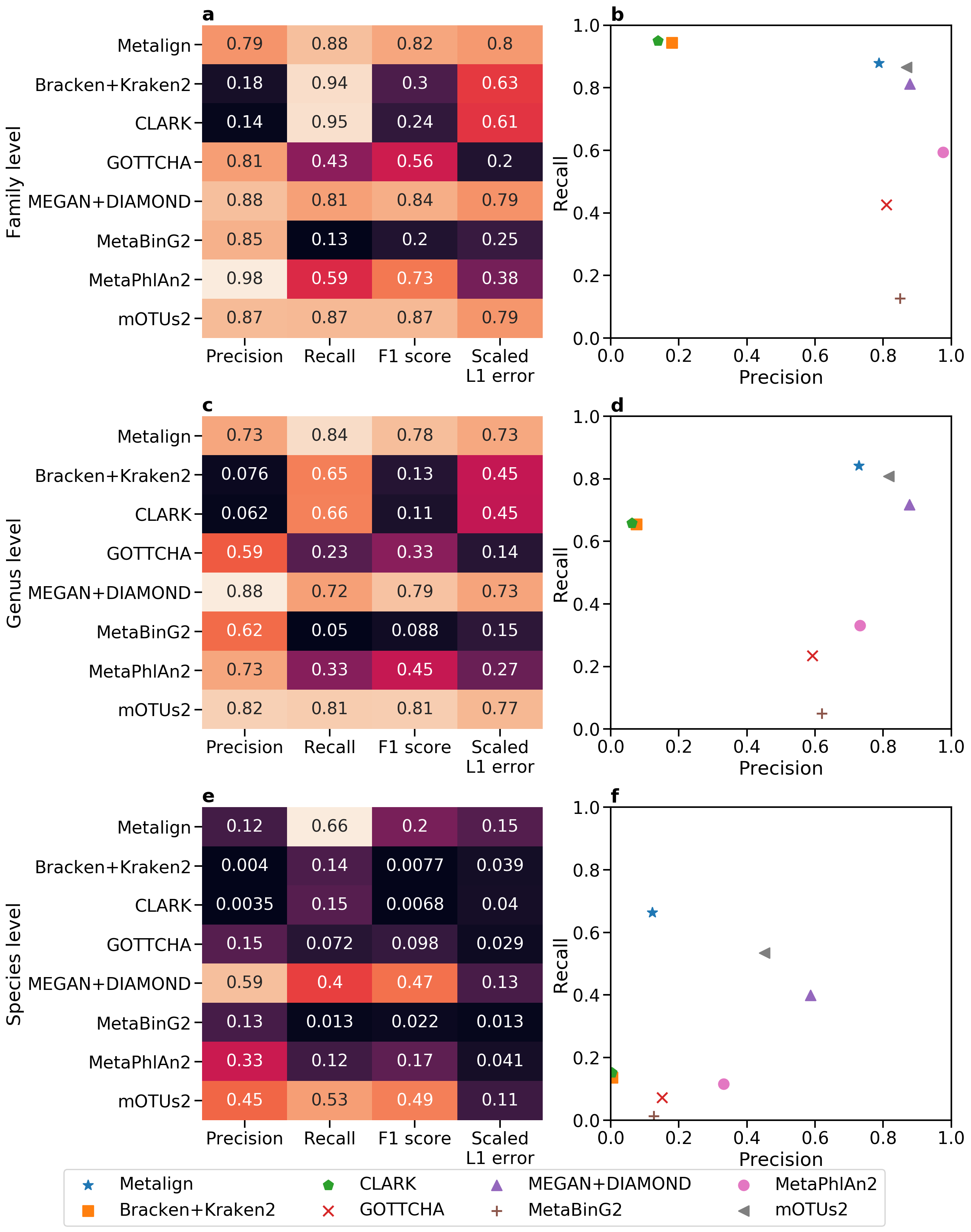

Supplement: Supplementary file 1 — Additional file 1. Supplementary text (including computing environments, information needed for replication, and performance metrics evaluated) and supplementary figures. [file 13059_2020_2159_MOESM1_ESM.zip › FigureS1.png]

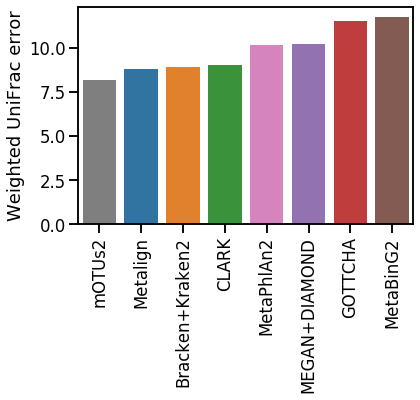

Supplement: Supplementary file 1 — Additional file 1. Supplementary text (including computing environments, information needed for replication, and performance metrics evaluated) and supplementary figures. [file 13059_2020_2159_MOESM1_ESM.zip › FigureS2.png]

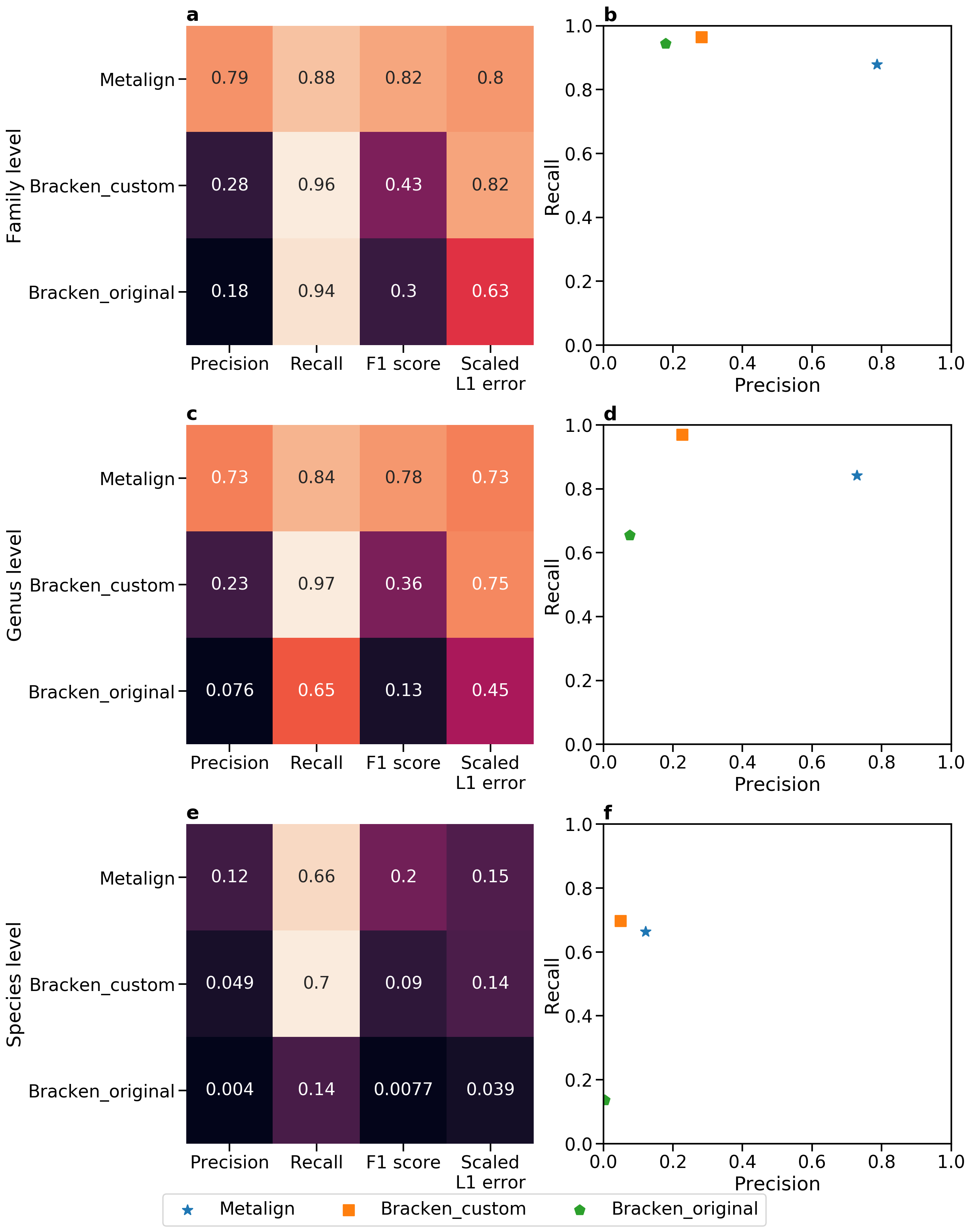

Supplement: Supplementary file 1 — Additional file 1. Supplementary text (including computing environments, information needed for replication, and performance metrics evaluated) and supplementary figures. [file 13059_2020_2159_MOESM1_ESM.zip › FigureS3.png]

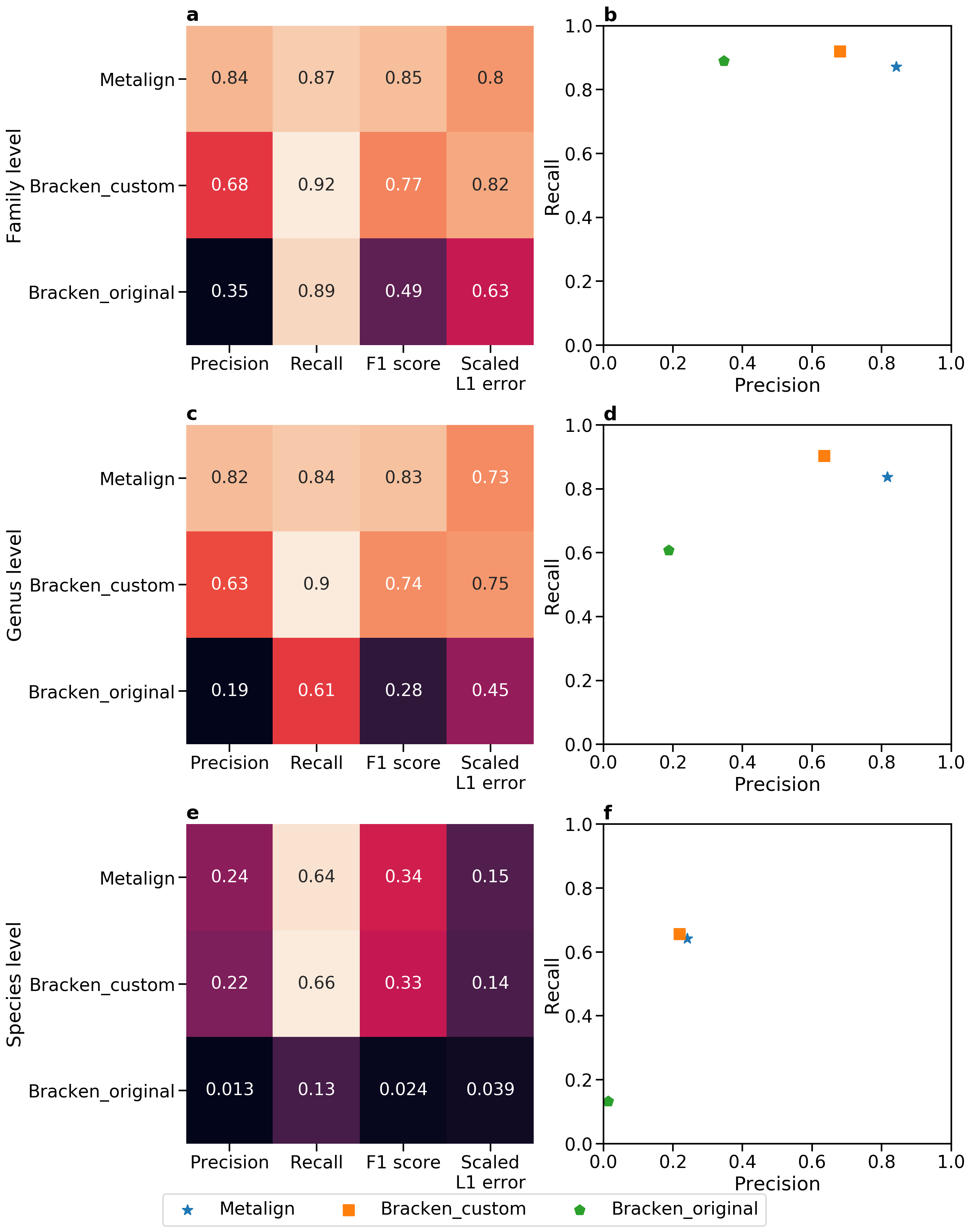

Supplement: Supplementary file 1 — Additional file 1. Supplementary text (including computing environments, information needed for replication, and performance metrics evaluated) and supplementary figures. [file 13059_2020_2159_MOESM1_ESM.zip › FigureS4.png]

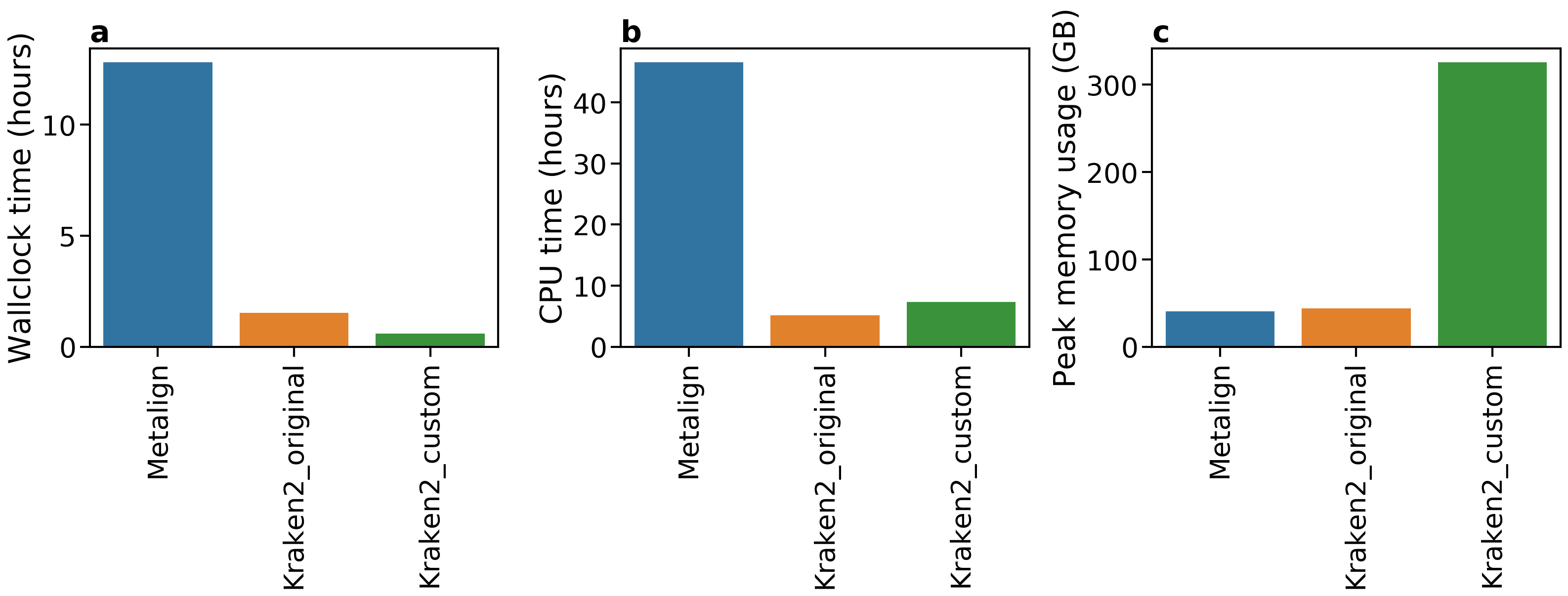

Supplement: Supplementary file 1 — Additional file 1. Supplementary text (including computing environments, information needed for replication, and performance metrics evaluated) and supplementary figures. [file 13059_2020_2159_MOESM1_ESM.zip › FigureS5.png]

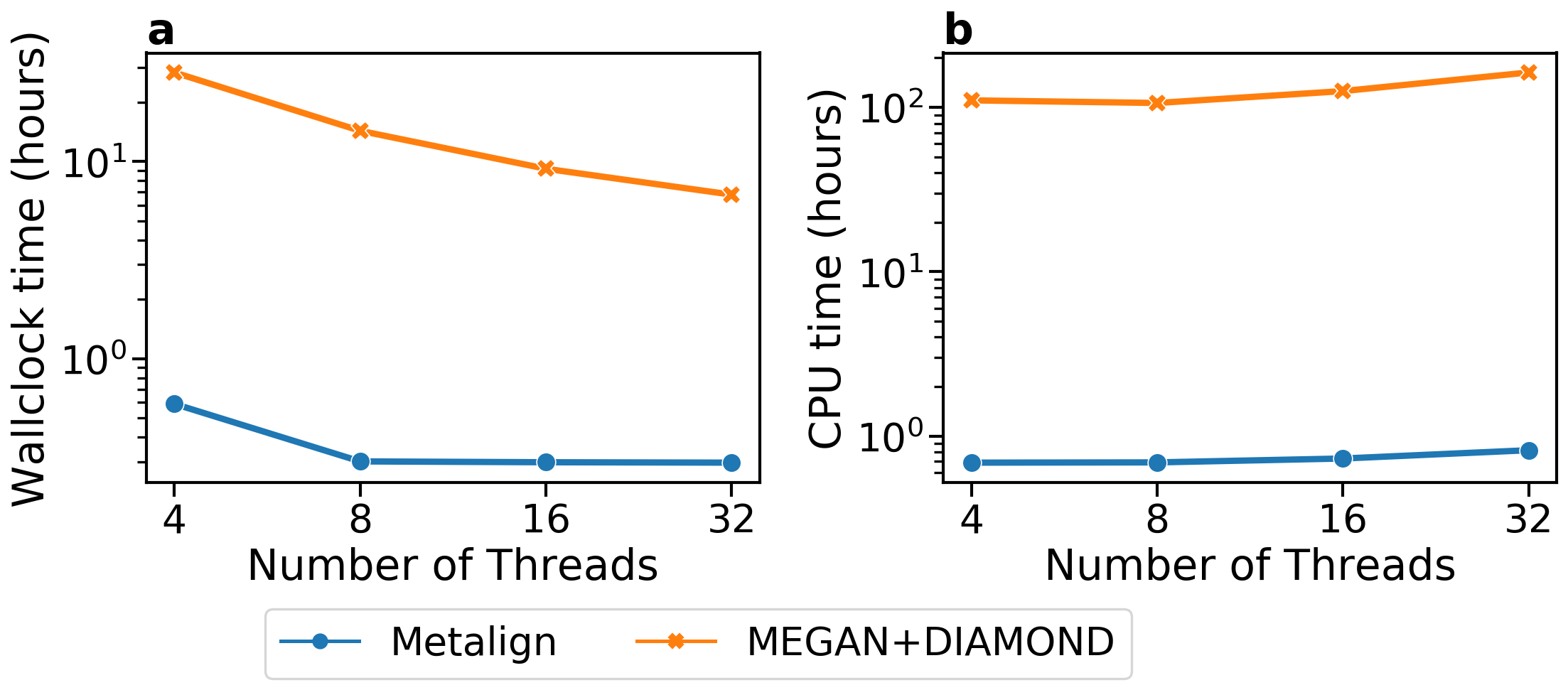

Supplement: Supplementary file 1 — Additional file 1. Supplementary text (including computing environments, information needed for replication, and performance metrics evaluated) and supplementary figures. [file 13059_2020_2159_MOESM1_ESM.zip › FigureS6.png]
