# Supplementary material for: Metalign: efficient alignment-based metagenomic profiling via containment min hash
Source: Genome Biol. 2020 Sep 10;21:242. doi: 10.1186/s13059-020-02159-0 (PMC7488264; doi:10.1186/s13059-020-02159-0)
Supplement: Supplementary file 2 — Additional file 2. Review history. [file 13059_2020_2159_MOESM2_ESM.docx]

Review History

**First round of review**

**Reviewer 1**

**Were you able to assess all statistics in the manuscript, including the appropriateness of statistical tests used?**

There are no statistics in the manuscript.

**Were you able to directly test the software?**

No

**Comments to author:**

Metagenomic profiling is a critical step in microbiome sequencing data analysis. Alignment based methods are accurate but computationally expensive. Sequence composition comparison based methods is fast but less accurate. The authors present here a novel alignment based methods which is fast and accurate by using containment min hash approach to reduce potential reference database size and a new read assignment method to deal with reads aligned to multiple reference genomes. A new tool called Metalign was constructed and validated with simulated datasets, mock community data and real world data. The pipeline can be a valuable tool for the research community. However, there are a large number of metagenome sequence classification, or profiling tools exist, only a few of them have been compared. There are some other issues with the analysis and results in the manuscript. Some of them are listed below.

Major revisions:

1.     Metagenomics profiling or organism abundance estimation in this paper is based on reference genome comparing, which means it is based on read classification, not binning. Although the classification result of each read can be discarded in the final abundance result, it is the initial or intermedium result of metagenomics profiling. Metagenomic sequence classification, binning and abundance estimation, these are three relevant but different concepts. The authors did not distinguish them clearly in the manuscript. For example, in page 23, line 42-47, "Note that this means that we do not perform explicit read classification/binning; rather, we try only to obtain the relative abundances of organisms, as accurately as possible".  I would suggest authors briefly distinct these three concept at the introduction.

2.     Using two or more rounds of abundance estimation can increase the specificity for metagenomics profiling methods. The abundance for each organism was estimated in the first round and the reads aligned to multiple organisms were assigned to the organism with highest frequency in the consequent round(s). Similar strategy has been reported by other metagenome sequence classification tools. This should be mentioned in the introduction or discussion.

3.     There are many metagenome sequencing classification tools can be used for metagenomics profiling. Why only MetaPhlAn2, Kraken2 and mOUTs2 were tested? How about KLARK and MetaBinG2?

4.     What is the memory and system requirement for Metalign?

5.     Increasing the cutoff threshold can also increase the specificity of other metagenomics profiling methods as the authors showed in Figure 3. It would be interesting to see the performance of the evaluated programs if the cutoff threshold was changed from percentages to read counts.  

6.     In page 17, before Figure 4, authors mentioned that "Metalign detects greater diversity of microbial communities in real world data". This is misleading, since in Fig 4c, the number of phyla found by Kraken2 was 20+3+6 = 29, which is greater than the number identified by Metalign only (7+3+3+6 = 19). Since the ground truth for real world data was unknown, therefore, it is not good for accuracy evaluation. The conclusion that "Metalign's results …. More comprehensive analyses of the data" was not well supported.

7.     Metalign can only use those reads that can be aligned to the reference database to estimate the organism abundance. In real world metagenomes, especially samples from ocean, only a small fraction of the sequencing reads can be aligned to the existing reference database. The performance of Metalign has not been evaluated for microbiomes with many unknown organisms. It would be interesting to see the performance of Metalign on sequencing data with a high percentage of reads unaligned to the reference database.

**Reviewer 2**

**Were you able to assess all statistics in the manuscript, including the appropriateness of statistical tests used?**

Yes, and I have assessed the statistics in my report.

**Were you able to directly test the methods?**

Yes

**Comments to author:**

LaPierre et al described Metalign, a software package for profiling metagenomic samples. Metalign combines three tools: KMC for k-mer counting, CMash for k-mer based filtering and minimap2 for read alignment. It uses a simple counting method to estimate the abundance of each genus/species. The authors show that on the simulated CAMI dataset, Metalign is  significantly more accurate at the genus level. Critical observations are as follows:

1) The authors are using a recent reference database and the CAMI results of other tools were submitted by 18 July 2015. The authors observed "re-running MetaPhlan2 using its most up-to-date reference database yielded similar results to its results in the original competition". Does "its up-to-date reference database" refer to the new marker gene database? When was that generated? What is the accuracy of MetaPhlAn2 on the new reference exactly? Even if MetaPhlAn2 doesn't benefit from a better reference database, other tools may benefit. I suggest the authors run Metalign on a version of reference database in 2015 for apple-to-apple comparisons; otherwise one can't confidently exclude the possibility that MetaPhlAn2's high accuracy comes from a more complete reference database.

2) I also wonder if the authors have selected the best tools for benchmarking. While MetaPhlAn2 seems overall the best in CAMI, it (and mOTUs2, both of which are marker based) performs poorly in a recent benchmark by Ye et al (2019) and is not as good as the traditional Blast+MEGAN at the genus level in McIntyre et al (2017). How is Metalign compared to Blast+MEGAN (or other alignment based approaches) and Kraken2/Bracken? The CAMI paper has evaluated these tools, but not for profiling. In general, I am surprised by the large differences between CAMI vs Ye/McIntyre et al. I worry the evaluation in the manuscript might not be representative.

3) Along this line, I wonder what enables the high accuracy of Metalign. Is it due to the use of complete reference genomes? It is important to analyze and understand the methodological advantages of Metalign, which can inspire further improvements in future.

4) I can run Metalign via docker. However, many "managed clusters" are not allowed to run docker. I strongly suggest the authors make it available via bioconda as well. It seems that Metalign doesn't have many dependencies and KMC and minimap2 are already available in bioconda. I guess porting to bioconda should not be very difficult.

Minor comments

5) I guess the authors are reporting wall clock time only. Please also report both CPU time.

6) It would be good to show both family- and species-level accuracy in the main paper. For example, the authors may move Fig S1a and S2a to Fig 2.

7) The authors claim that "the Kraken family of methods is well-known to generate a number of very low abundance false positives". However, I couldn't find the source. Peabody et al only shows unfiltered Kraken has false positives, but it doesn't mention the "Kraken family". The CAMI paper is not using Kraken for profiling.

Reviewer: Heng Li

**Authors Response**

**Point-by-point responses to the reviewers’ comments:**

*Response*

**Response to Reviewers' Comments**

Reviewer 1 Remarks to the Author:

Metagenomic profiling is a critical step in microbiome sequencing data analysis. Alignment based methods are accurate but computationally expensive. Sequence composition comparison based methods is fast but less accurate. The authors present here a novel alignment based methods which is fast and accurate by using containment min hash approach to reduce potential reference database size and a new read assignment method to deal with reads aligned to multiple reference genomes. A new tool called Metalign was constructed and validated with simulated datasets, mock community data and real world data. The pipeline can be a valuable tool for the research community. However, there are a large number of metagenome sequence classification, or profiling tools exist, only a few of them have been compared. There are some other issues with the analysis and results in the manuscript. Some of them are listed below.

Reviewer 1 Specific Comment #1:

Metagenomics profiling or organism abundance estimation in this paper is based on reference genome comparing, which means it is based on read classification, not binning. Although the classification result of each read can be discarded in the final abundance result, it is the initial or intermedium result of metagenomics profiling. Metagenomic sequence classification, binning and abundance estimation, these are three relevant but different concepts. The authors did not distinguish them clearly in the manuscript. For example, in page 23, line 42-47, "Note that this means that we do not perform explicit read classification/binning; rather, we try only to obtain the relative abundances of organisms, as accurately as possible". I would suggest authors briefly distinct these three concept at the introduction.

*Author Response:*

*We thank the reviewer for noting the importance of making these distinctions. We have now in the introduction defined and briefly discussed metagenomic assembly, read classification, taxonomic read binning, and taxonomic profiling (which includes abundance estimation). The text now reads as follows, with modified portions in red:*

*Predicting the presence and relative abundance of taxa in a metagenomic sample (referred to as "taxonomic profiling") is one of the primary means of analyzing a metagenomic sample* *7,8. In comparison to metagenomic assembly, profiling is computationally simpler and more effective at identifying low-abundance organisms* *8. Metagenomic profiles can be obtained through read classification (where individual reads are assigned to taxa or*

*organisms) or via the closely related technique of read binning (grouping individual reads into putative single taxa or organism groups). However, due to the necessary step of inferring taxa from individual short reads, these strategies have been shown to produce less accurate taxonomic profiles* [*7*](https://paperpile.com/c/hYEvAp/nyB4O)*.*

Reviewer 1 Specific Comment #2:

Using two or more rounds of abundance estimation can increase the specificity for metagenomics profiling methods. The abundance for each organism was estimated in the first round and the reads aligned to multiple organisms were assigned to the organism with highest frequency in the consequent round(s). Similar strategy has been reported by other metagenome sequence classification tools. This should be mentioned in the introduction or discussion.

*Author Response:*

*We thank the reviewer for this suggestion of a future improvement to Metalign, so we have mentioned this in the Discussion section and cited related literature on this topic. The Discussion section now contains the text:*

*Additionally, an iterative, Bayesian-like approach could leverage an initial taxonomic profile produced by Metalign as a prior upon subsequent re-assignment of multi-mapped reads. Similar Bayesian-like approaches are useful for related metagenomic analysis task*[*s*](https://paperpile.com/c/hYEvAp/nCVq)*34.*

Reviewer 1 Specific Comment #3:

There are many metagenome sequencing classification tools can be used for metagenomics profiling. Why only MetaPhlAn2, Kraken2 and mOUTs2 were tested? How about KLARK and MetaBinG2?

*Author Response:*

*Thank you for bringing our attention to more methods we should run. We have added CLARK and MetaBinG2 to the experiments, as well as MEGAN and GOTTCHA due to the reviewer 2 specific comment #2. We also added Bracken+Kraken2 and mOTUs2 to the CAMI experiment, and ran MetaPhlAn2 with its most up-to-date database instead of using the published CAMI results. Please see the updated Figures 2 & 4. We also added a new results section to summarize our findings, under the header “Metalign advantages compared to top-performing methods” (excluded here due to its length).*

Reviewer 1 Specific Comment #4:

What is the memory and system requirement for Metalign?

*Author Response:*

*We thank the reviewer for bringing our attention to this. We have added Figures 3 and 5, showing the wallclock time, CPU time, and memory cost for all methods on the CAMI data and real data, respectively. We have now ported Metalign to Bioconda, so it is now platform-independent. The relevant text is below:*

*Metalign is freely available at* [*https://github.com/nlapier2/Metalign*](https://and) *and a Bioconda package is available at* [*https://anaconda.org/bioconda/metalign*](https://anaconda.org/bioconda/metalign)*.*

*{...}*

*Meanwhile, CLARK had very high memory requirements (157.6 GB peak memory usage), while MetaBinG2, MetaPhlAn2, and mOTUs2 had very low memory requirements (less than 2.5 GB). GOTTCHA had a peak memory usage of 10.5 GB; Metalign, Kraken2, and MEGAN+DIAMOND used 40-44 GB (Figure 3c).*

*{...}*

*We also used real data to benchmark the methods in terms of wallclock runtime, CPU time, and peak memory usage on more realistic data (Figure 5). We used two datasets from the Tara Oceans project* [*16*](https://paperpile.com/c/hYEvAp/cKCBC) *in order to evaluate the scaling performance of the methods from the small ~0.1 GB Peabody dataset to 11 GB and 98 GB datasets from the Tara Oceans project* [*16*](https://paperpile.com/c/hYEvAp/cKCBC)*. The reads originally came in separate gzipped paired end files, which we decompressed and then interleaved with BBMap [32](https://paperpile.com/c/hYEvAp/rreOM) before running the methods on this data.*

*{...}*

*In terms of memory usage, CLARK remained the highest by far while MetaPhlAn2, mOTUs2, and MetaBinG2 were the lowest and the rest of the methods were in-between. Most methods used roughly the same amount of memory regardless of file size, except CLARK, whose memory usage increased sub-linearly with the size of the input file.*

Reviewer 1 Specific Comment #5:

Increasing the cutoff threshold can also increase the specificity of other metagenomics profiling methods as the authors showed in Figure 3. It would be interesting to see the performance of the

evaluated programs if the cutoff threshold was changed from percentages to read counts.

**Author response:**

We thank the reviewer for this idea. It would indeed be interesting to see how performance would change when setting the cutoff to read counts. Unfortunately, not all taxonomic profiling methods utilize read counts to infer their taxonomic profile. Indeed, Kraken2 utilizes k-mer information, MetaPhlAn2 utilizes marker gene information, and MetaBinG2 uses a k-mer-based similarity approach. As such, we are unable to change the cutoff threshold from percentages to read counts.

Reviewer 1 Specific Comment #6:

In page 17, before Figure 4, authors mentioned that "Metalign detects greater diversity of microbial communities in real world data". This is misleading, since in Fig 4c, the number of phyla found by Kraken2 was 20+3+6 = 29, which is greater than the number identified by Metalign only (7+3+3+6 = 19). Since the ground truth for real world data was unknown, therefore, it is not good for accuracy evaluation. The conclusion that "Metalign's results .... More comprehensive analyses of the data" was not well supported.

*Author response:*

*We thank the reviewer for bringing this to our attention. We agree with the reviewer about the evaluation not being usable due to the lack of ground truth. We have decided to remove all performance evaluations of the Tara Oceans results. Instead, we now only use this data to benchmark runtime and memory performance on real data (as discussed in response to the next comment).*

Reviewer 1 Specific Comment #7:

Metalign can only use those reads that can be aligned to the reference database to estimate the organism abundance. In real world metagenomes, especially samples from ocean, only a small fraction of the sequencing reads can be aligned to the existing reference database. The performance of Metalign has not been evaluated for microbiomes with many unknown organisms. It would be interesting to see the performance of Metalign on sequencing data with a high percentage of reads unaligned to the reference database.

*Author response:*

*As the reviewer correctly points out, reference-based taxonomic profiling techniques can all suffer in performance when presented with samples that contain organisms not present in their reference database. However, as Metalign only aims to produce a taxonomic profile, we have found that (due to sequence homology and potentially high ANI between the novel organism(s) and reference organisms), Metalign can still correctly infer higher taxonomic ranks for novel organisms. Indeed, on the first CAMI high complexity benchmark dataset (which was designed by CAMI specifically to contain many novel organisms), Metalign’s reference database has only 162 reference organisms that matched the species taxID’s out of a total of 243 unique species taxID’s in this dataset. Additionally, a high portion of the total sample abundance is composed of unmappable reads; for instance, in the aforementioned dataset, over 71.4% of the sample abundance consists of novel strains. Nonetheless, as demonstrated in Fig. 2, Metalign was still successful at profiling this sample due to the aforementioned sequence homology etc. We have drawn out this point in the main text to clarify that the benchmarking was indeed performed on datasets that contained organisms not exactly represented in Metalign’s training database. We have added the following text to the paper:*

*Of note, the high complexity communities contained many organisms not represented in*

*the Metalign training database. For example, in one of the CAMI high complexity datasets, Metalign’s database contained only 161 out of the 243 unique species in the dataset. Additionally, a high percentage of the total sample abundance is composed of unmappable reads; for example, in the aforementioned dataset, over 71.4% of the sample abundance consists of novel strains.*

Reviewer 2 Remarks to the Author:

LaPierre et al described Metalign, a software package for profiling metagenomic samples. Metalign combines three tools: KMC for k-mer counting, CMash for k-mer based filtering and minimap2 for read alignment. It uses a simple counting method to estimate the abundance of each genus/species. The authors show that on the simulated CAMI dataset, Metalign is significantly more accurate at the genus level. Critical observations are as follows:

Reviewer 2 Specific Comment #1:

The authors are using a recent reference database and the CAMI results of other tools were submitted by 18 July 2015. The authors observed "re-running MetaPhlan2 using its most up-to-date reference database yielded similar results to its results in the original competition". Does "its up-to-date reference database" refer to the new marker gene database? When was that generated? What is the accuracy of MetaPhlAn2 on the new reference exactly? Even if MetaPhlAn2 doesn't benefit from a better reference database, other tools may benefit. I suggest the authors run Metalign on a version of reference database in 2015 for apple-to-apple comparisons; otherwise one can't confidently exclude the possibility that MetaPhlAn2's high accuracy comes from a more complete reference database.

*Author response:*

*We appreciate this feedback and as such, for MetaPhlAn2 and all other methods we ran (including the ones we added; see below), we stopped using the 2015 results, and instead we ran all methods with their most up-to-date default reference databases available as of April 2020. Since the most recent default reference database is by far the most common use case for each of these methods, we think this is the most realistic and practical comparison. We now say the following in the text (changes in red):*

*We ran Metalign on these eight datasets and compared our results with several other state-of-the-art methods: Kraken2* [*20*](https://paperpile.com/c/hYEvAp/TNwC) *with its abundance estimation companion method Bracken* [*21*](https://paperpile.com/c/hYEvAp/8EP3) *(“Bracken+Kraken2”), CLARK* [*22*](https://paperpile.com/c/hYEvAp/bbyf)*, MetaPhlAn2* [*23*](https://paperpile.com/c/hYEvAp/EUeH)*, mOTUs2* [*24*](https://paperpile.com/c/hYEvAp/uRBY)*, MetaBinG2* [*25*](https://paperpile.com/c/hYEvAp/z3YD)*, GOTTCHA* [*26*](https://paperpile.com/c/hYEvAp/y3Q3)*, and MEGAN6* [*27,28*](https://paperpile.com/c/hYEvAp/mrFr+N4UY) *with its associated fast alignment method DIAMOND* [*29*](https://paperpile.com/c/hYEvAp/07Kz) *(“MEGAN+DIAMOND”). The first four of these methods were chosen for their wide usage and past involvement with the CAMI competition, and the latter two were added because of their strong performance on benchmarking studies by McIntyre et al.* [*22*](https://paperpile.com/c/hYEvAp/bbyf) *and Ye et al.* [*30*](https://paperpile.com/c/hYEvAp/DZ7m)*.*

*The comparisons were performed using the CAMI-affiliated evaluation software OPAL* [*17*](https://paperpile.com/c/hYEvAp/nPL9j) *(Figure 2), and timing and memory usage statistics were collected (Figure 3). Each method was run with its default database; CLARK was set to detect organisms from all available taxonomic ranks.*

*Additionally, we added another experiment to facilitate an “apples-to-apples” comparison. Since Kraken2 is one of the few methods tested that can straightforwardly be re-trained on a custom reference database, we re-trained it using the exact same database that Metalign uses. This significantly improved the precision and F1 score of Bracken+Kraken2; however, these values were still lower than those of Metalign. Additionally, Kraken2’s memory usage with our comprehensive database spiked to over 325 GB, making this impractical for most users. Metalign is able to avoid this high memory usage due to CMash’s lightweight “sketch”-based data structures and the fact that alignment doesn’t require so much memory. The relevant text reads:*

*We repeated this experiment with Bracken+Kraken2 using the same custom database as Metalign with no abundance cutoff (Figure S3) and with a 0.01% abundance cutoff (Figure S4). Using Metalign’s custom database, Kraken2 achieved much better precision than with its original database, but Kraken2’s precision and F1 score were still lower across all taxonomic ranks evaluated and both abundance cutoff settings when compared to Metalign. For example, Kraken2’s precision at the genus level using a 0.01% abundance cutoff increased to 63.5% compared with 18.7% using its original database but was still lower than Metalign’s 81.7% precision; similarly, its F1 score increased from 27.6% to 73.5% compared with Metalign’s 82.5%. Importantly, in terms of memory usage (Figure S5), Kraken2 peaked at 325.23 GB with the custom database, suggesting that the use of such a comprehensive database would be infeasible for many users. In comparison, Metalign’s peak memory usage was 40.37 GB.*

Reviewer 2 Specific Comment #2:

I also wonder if the authors have selected the best tools for benchmarking. While MetaPhlAn2 seems overall the best in CAMI, it (and mOTUs2, both of which are marker based) performs poorly in a recent benchmark by Ye et al (2019) and is not as good as the traditional Blast+MEGAN at the genus level in McIntyre et al (2017). How is Metalign compared to Blast+MEGAN (or other alignment based approaches) and Kraken2/Bracken? The CAMI paper has evaluated these tools, but not for profiling. In general, I am surprised by the large differences between CAMI vs Ye/McIntyre et al. I worry the evaluation in the manuscript might not be representative.

**Author Response:**

We appreciate the reviewer drawing our attention to this, and as such have run significantly more tools (including those suggested by the reviewer). We were unable to run MEGAN+BLAST on the CAMI datasets due to job timeouts on our server after a week of running time, but instead ran MEGAN+DIAMOND which, according to the McIntyre et al review, seemed to perform comparably to MEGAN+BLAST but with much better runtime. We also included GOTTCHA because it performed well in presence/absence prediction in the McIntyre study, as well as CLARK and MetaBinG2 according to the feedback of the other reviewer. For both the CAMI data (Figure 2) and real data (Figure 4), we now run the following tools: Metalign, Bracken+Kraken2, CLARK, GOTTCHA, MEGAN+DIAMOND, MetaBinG2, MetaPhlAn2, and mOTUs2.

*It appears that the results we obtained in these updated experiments largely recapitulated the general conclusions of the Sczyrba et al (CAMI), McIntyre et al, Peabody et al, and Ye et al studies, and made an effort to mention and cite in the results sections every time this was the case. For instance, examining Figures 2 and 3:*

*MEGAN+DIAMOND was highly accurate but slow (McIntyre et al, Ye et al)*

*Bracken+Kraken2 and CLARK had high recall and fast speed but poor precision (Sczyrba et al, McIntyre et al, Peabody et al)*

*GOTTCHA had strong precision but poor abundance estimation (McIntyre et al)*

*MetaPhlAn2 had strong precision but poor recall and abundance estimation (Sczyrba et al, McIntyre et al)*

*mOTUs2 performs well on CAMI simulations (Milanese et al, personal communication with the CAMI2 team) but poorly on some other datasets (Ye et al).*

*Here is the updated text (relevant citations were re-formatted for this letter):*

*The comparisons were performed using the CAMI-affiliated evaluation software OPAL*

[*17*](https://paperpile.com/c/hYEvAp/nPL9j) *(Figure 2), and timing and memory usage statistics were collected (Figure 3). {...}*

*Metalign, MEGAN+DIAMOND, and mOTUs2 substantially outperformed Bracken+Kraken2, CLARK, GOTTCHA, MetaBinG2 and MetaPhlan2 in both presence/absence prediction (F1 Score) and abundance estimation (L1 error). As expected from previous studies* [*7,15,22*](https://paperpile.com/c/hYEvAp/nyB4O+bbyf+nQ7qN)*, Bracken+Kraken2 and CLARK produce results with high false positive rates. However, regardless of the abundance cutoff threshold chosen, these methods produce an F1 score that falls short of the top-performing methods. MetaPhlan2 and GOTTCHA produce results with strong precision but comparatively high false negative rate and abundance estimation error, as noted in previous studies* [*7,22*](https://paperpile.com/c/hYEvAp/nyB4O+bbyf)*. MetaBinG2, like GOTTCHA, had good precision but poor recall and abundance estimation. Metalign, MEGAN+DIAMOND, and mOTUs2 performed similarly on most performance metrics, except Weighted UniFrac, where mOTUs2 outperformed all other methods. Metalign is the next-best method, and MEGAN+DIAMOND ranks sixth out of the eight methods assessed (Figure S2).*

*Metalign ranked near the middle of the methods in terms of runtime and memory. As demonstrated by previous benchmarking studies* [*22,30*](https://paperpile.com/c/hYEvAp/DZ7m+bbyf)*, MEGAN+DIAMOND was much slower than all other methods (Figure 3a-b), taking 2072.3 wall clock hours while no other method took more than 15 hours. Kraken2 was the fastest method (1.5 wall clock hours to run on all datasets), followed by CLARK (6.7 hours), with all other methods taking between 9 and 15 wall clock hours (Figure 3a-b). Meanwhile, CLARK had very high memory requirements (157.6 GB peak memory usage), while MetaBinG2, MetaPhlAn2, and mOTUs2 had very low memory requirements (less than 2.5 GB).*

*GOTTCHA had a peak memory usage of 10.5 GB; Metalign, Kraken2, and MEGAN+DIAMOND used 40-44 GB (Figure 3c).*

*{...}*

*mOTUs2 has previously been shown to perform well on the CAMI data (Milanese et al)*

*but less well on other datasets (Ye et al), and our results suggest a similar inconsistency.*

*We also added a new results section to summarize our findings, under the header “Metalign advantages compared to top-performing methods” (excluded here due to its length).*

*Citations:*

*Milanese A, Mende DR, Paoli L, Salazar G, Ruscheweyh HJ, Cuenca M, Hingamp P, Alves R, Costea PI, Coelho LP, Schmidt TS. Microbial abundance, activity and population genomic profiling with mOTUs2. Nature communications. 2019 Mar 4;10(1):1-1.*

*McIntyre AB, Ounit R, Afshinnekoo E, Prill RJ, Hénaff E, Alexander N, Minot SS, Danko D, Foox J, Ahsanuddin S, Tighe S. Comprehensive benchmarking and ensemble approaches for metagenomic classifiers. Genome biology. 2017 Dec;18(1):182.*

*Sczyrba, Alexander, Peter Hofmann, Peter Belmann, David Koslicki, Stefan Janssen, Johannes Dröge, Ivan Gregor, et al. 2017. “Critical Assessment of Metagenome Interpretation—a Benchmark of Metagenomics Software.” Nature Methods 14 (11): 1063–71.*

*Ye SH, Siddle KJ, Park DJ, Sabeti PC. Benchmarking metagenomics tools for taxonomic classification. Cell. 2019 Aug 8;178(4):779-94.*

Reviewer 2 Specific Comment #3:

Along this line, I wonder what enables the high accuracy of Metalign. Is it due to the use of complete reference genomes? It is important to analyze and understand the methodological advantages of Metalign, which can inspire further improvements in future.

*Author Response:*

*We appreciate this feedback from the reviewer, and as such, we have added some analysis to help explain the accuracy of Metalign. From our analyses, it appears that the high accuracy of Metalign is due to two aspects:*

*Primarily, the initial database reduction step significantly reduces the possibility of false positives by removing from the training database those organisms that cannot reasonably be expected to be present in the sample (due to low containment index with the sample). If these reference genomes were not removed, accurate downstream alignment can be impeded by the inclusion of such organisms due to read alignments present merely due to homology.*

*Secondarily, the treatment of uniquely mapped versus multi-mapped reads further increases the abundance estimation accuracy of Metalign. Note that the number of uniquely mapped reads can only decrease (thus decreasing accuracy) if the database reduction step is not performed.*

*We have significantly revised the discussion section to mention this and other aspects of Metalign that could inspire further improvements or analyses in the future. The relevant portion of the discussion reads as follows:*

*We developed Metalign, a novel computational approach capable of achieving a strong*

*balance between precision and recall across a variety of datasets, community diversities, and taxonomic levels. Several factors drive Metalign’s high performance. Metalign’s novel pre-filtering, database reduction step substantially quickens and increases the precision of downstream alignment and taxonomic profile inference due to the removal of organisms that cannot reasonably be contained in the sample (due to low containment index) but would instead just confound sequence alignment efforts. Metalign is able to obtain high precision because it requires an organism to pass the pre-filtering threshold and have at least one high-quality read mapping uniquely to its genome in the subsequent alignment stage. At the same time, Metalign maintains high recall by using a relatively liberal pre-filtering threshold that only excludes organisms which cannot reasonably be present, and by not imposing excessive read alignment or genome coverage requirements in the mapping stage. Abundance estimation is improved by considering multi-aligned reads.*

*The pre-filtering technique will continue to enable alignment-based metagenomic analysis techniques even as reference databases continue to grow in size. Using this approach, alignment is only performed on the portions of the reference database that are relevant to the input metagenomic sample. Given the speed and accuracy results, the resulting alignments of Metalign could be used for other analyses (e.g. microbial genome rearrangement, microbial Genome-Wide Association Studies, etc.) that would not be practically possible with a standard sequence aligner on the full database (as demonstrated by the run-time results depicted for DIAMOND in Figure 5 and BLAST in Figure 1). Additionally, Metalign’s database can be updated and re-trained in a straightforward manner, in contrast to marker gene based methods such as MetaPhlAn2 and the alignment-based mOTUs2* [*30*](https://paperpile.com/c/hYEvAp/DZ7m) *which uses bespoke constructed metagenomic OTU’s derived from real metagenomic samples.*

Reviewer 2 Specific Comment #4:

I can run Metalign via docker. However, many "managed clusters" are not allowed to run docker. I strongly suggest the authors make it available via bioconda as well. It seems that Metalign doesn't have many dependencies and KMC and minimap2 are already available in bioconda. I guess porting to bioconda should not be very difficult.

**Author Response:**

We too can appreciate the difficulty of running docker on a managed cluster. As such, we have ported

Metalign to Bioconda, so it is now platform-independent and the issues with Docker can be avoided. We

now mention this in the abstract (new text in red):

Metalign is freely available at [https://github.com/nlapier2/Metalign](https://and) and a Bioconda

package is available at <https://anaconda.org/bioconda/metalign>.

Reviewer 2 Specific Comment #5:

Minor comments

5) I guess the authors are reporting wall clock time only. Please also report both CPU time.

**Author Response:**

We thank the reviewer for bringing our attention to this. We have added Figures 3 and 5, showing the wallclock time, CPU time, and memory cost for all methods on the CAMI data and real data, respectively. DIAMOND timed out after 168 wallclock hours (1 week) on our server for the largest Tara Oceans dataset, which is why that value is missing from Figure 5.

Reviewer 2 Specific Comment #6:

It would be good to show both family- and species-level accuracy in the main paper. For

example, the authors may move Fig S1a and S2a to Fig 2.

**Author Response:**

We thank the reviewer for this suggestion. As requested, we have added family-level and species-level performance to the main paper (see Figure 2). In order to avoid an oversized figure, we now only display the heatmap and precision/recall scatterplots for each level.

Reviewer 2 Specific Comment #7:

The authors claim that "the Kraken family of methods is well-known to generate a number of very low abundance false positives". However, I couldn't find the source. Peabody et al only shows unfiltered Kraken has false positives, but it doesn't mention the "Kraken family". The CAMI paper is not using Kraken for profiling.

Reviewer: Heng Li

*Author Response:*

*We thank the reviewer for raising this issue. We now avoid using the phrase “Kraken family”. Instead, we simply cite past studies (e.g. Sczyrba et al, Peabody et al, McIntyre et al) that have mentioned and/or demonstrated that Kraken and CLARK generate many low-abundance false positives, so a cutoff threshold is often employed. We also have a figure that demonstrates this phenomenon on the CAMI data. We have added the following text and figure:*

*Because methods such as CLARK and Kraken2 are known to produce a large number of*

*low-abundance false positives* [*7,15,22*](https://paperpile.com/c/hYEvAp/nyB4O+nQ7qN+bbyf)*, we removed organisms assigned less than 0.01% abundance from the profiles of all methods. This cutoff threshold resulted in a better F1 score for these methods than not using a threshold (Figure S1). Further details on how the tools were run are available in the Supplementary Information.*

**Updated / New Figures**

0.87

mOTUs2 -

0.85

**0.79**

0.86

0.82

0.84

0.83

0.88

0.72

0.79

0.73

0.05

0.088	0.15

0.29

0.42

0.27

0.82

0.77

0.79

0.84

Metalign -

Bracken +Kraken2

CLARK

GOTTCHA

a) = MEGAN+DIAMOND -

MetaBinG2 MetaPhlAn2 - mOTUs2 -

Precision

**I	I**	0.0	**I	I**	I

Recall	Fl score	Scaled	0 0	0.2	0.4	0.6	0.8

Ll error	Precision

1.0 **^d^**

0.73

10

0.8 -

0.6 -

=

fa

a)

0.4 -

0.2 -

1.0

***_4_**

**•**

0.8 -

•

0.6 -

Ta a)

x

0.4 -

0.2 -

a

Metalign -

Bracken+Kraken2 -

CLARK-

(7.)

a.)	GOTTCHA-

_

>,

E MEGAN+DIAMOND -

ID

**U-**

MetaBinG2 -

MetaPhlAn2 -

**0.62**

0.75

0.81

0.84

0.25

0.13	0.2

0.54

01^1^:9 ^mr-^	0.38

0.8

0.85

**0.49	0.63**

**0.62**	0.62

0.56	0.2

0.84

0.87

0.89

0.88

0.85

0.98

*

**4**

•

•

•

x

0.0	i

Precision	Recall^i^	Fl score	Scaled	0 0	0.2	0.4	0.6	0.8

10

Li error	Precision

0.15

0.039

0.04

0.029

**_L_** 0.13

0.013

0.041

0.11

Sca led Ll error

e

| a) a) | Metalign - Bracken+Kraken2 - CLARK-GOTTCHA- | 0.24 | 0.64 | 0.34  0.024  0.072  0.098 |
| --- | --- | --- | --- | --- |
|  |  | 0.013  0.053 | 0.13  0.13 |  |
|  |  | 0.15 | 0.072 |  |
| a) MEGAN+DIAMOND- | | 0.59 | **0** |  |
| Q.  *(r)* |  |  |  |  |
|  | MetaBinG2 - | 0.13 | **0.013** | **0.022** |
|  | MetaPhlAn2 - | 0.34 | 0.1 | 0.15 |
|  | mOTUs2 - | 0s. I | 0.53 |  |
|  |  | Precision | Recall | Fl score |

| 1.0  0.8 - | | | |
| --- | --- | --- | --- |
|  |  |  |  |
|  |  |  |  |
|  |  | * |  |
|  |  |  | **4** |
| 0.4 - | |  | • |
|  |  |  |  |
| 0.2 - | |  |  |
|  |  | x |  |
|  |  |  |  |

0.0

0 0	0.2	0.4	0.6

Precision

0.8	10

* Metalign	• CLARK	• MEGAN+DIAMOND	MetaPhlAn2

- Bracken+Kraken2 x GOTICHA + MetaBinG2	i mOTUs2

*Figure 2. Comparison of Metalign with competing methods on the challenge datasets from the first CAMI competition with organisms below 0.01% relative abundance excluded. Heatmaps show Precision, Recall, F1 score, and L1 error re-scaled such that higher is better (1 - (L1 error / 2)) at the (a) family level, (c) genus level, and (e) species level. Scatter plots show Precision (x-axis) versus Recall (y-axis) at the (b) family level,* *(d) genus level, and (f) species level. Metrics were averaged across all eight datasets.*

*Figure 3. Comparison of Metalign with competing methods on the challenge datasets from the first CAMI competition in (a) wallclock running time, (b) CPU time, and (c) peak memory usage in gigabytes. The running times were summed across all eight datasets, while the memory values reflect the peak memory usage across all eight datasets.*

*Figure 4. Genus-level F1 score at different abundance cutoff thresholds using in vitro mock community from Peabody et al. 2015* *15. For a given cutoff threshold, we calculated the F1 score while leaving out predicted genera below the threshold.*

*Figure 5. (a) Wall clock running time in hours, (b) CPU time in hours, and (c) Peak memory usage in gigabytes of the tested methods on three real datasets of various sizes: the Peabody mock community (0.1 GB) and two samples from the Tara Oceans project (11 GB and 98 GB). In order to generate comparable files, the Peabody data was converted to FASTQ format and the separate paired-end and gzipped Tara Oceans files were decompressed and interleaved. MEGAN+DIAMOND did not finish running after 168 wallclock hours (one week) on the 98 GB file.*

* Metalign

- CLARK	• MEGAN+DIAMOND	MetaPhlAn2

o Bracken+Kraken2 x GOTTCHA + MetaBinG2	**1** mOTUs2

I	I	I	I

I	I	I

Precision	Recall	Fl score	Scaled	0 0	0.2	0.4	0.6	0.8

Ll error	Precision

**c	d**

0.84

0.78

0.82

0.77

0.81

0.81

Metalign -

Bracken+Kraken2 -

CLARK-

a)	GOTTCHA-

_

a) c MEGAN+DIAMOND -

0.73

0.73

0.88

0.72

0.79

0.73

MetaBinG2-MetaPhlAn2-mOTUs2 -

0.45

0.45

0.0

Precision	Recall^i^	Fl s^1^core	Scaled	0 0	0!2	0.4	0.6	^1^ 0.8

Ll error	Precision

0.0

0.8 -

**1.0**

*

I

0.4 -

x

0.2 -

10

•

10

0.8

0.82

| **a** | |
| --- | --- |
| Metalign - | 0.79 |
| Bracken+Kraken2 - | 0.18 |
| CLARK- | 0.14 |
| U) GOTTCHA- | 0.81 |
| **E** MEGAN+DIAMOND - u_ | 0.88 |
| MetaBinG2- | 0.85 |
| MetaPhlAn2- | 0.98 |
| mOTUs2 - | 0.87 |

0.88

0.81

0.84

0.79

0.87

0.87

0.79

0.63

0.61

**1.0**

•

I

•

0.8

0.6 -

=

x

0.4

0.2 -

e

| Metalign - | 0.12 | 0.66 | 0.2 |
| --- | --- | --- | --- |
| Bracken+Kraken2 - | 0.004 | 0.14 | 0.0077 |
| CLARK- | 0.0035 | 0.15 | 0.0068 |
| GOTTCHA- | 0.15 | 0.072 | 0.098 |
| 'D MEGAN+DIAMOND-  U)  **a.** | 0.59 | 0.4 | 0.47 |
| MetaBinG2- | 0.13 | 0.013 | 0.022 |
| MetaPhlAn2- | 0.33 | **0.**12 | 0.17 |
| mOTUs2 - | 0.45 |  | 0.49 |
| Precision | | Recall | Fl **score** |

x

**1.0**

**0.8-**

*

I

0.4 -

•

0.2 -

0.8

10

0.0

0 0	0.2	0.4	0.6

Precision

0.15 0.039 0.04 0.029 0.13

0.013

0.041

0.11

Scaled

Li error

*Figure S1. Comparison of Metalign and competing methods when applied to the challenge datasets from the first CAMI competition with no abundance threshold. Heatmaps show Precision, Recall, F1 score, and L1 error re-scaled such that higher is better (1 - (L1 error / 2)) at the (a) family level, (c) genus level, and (e) species level. Scatter plots show Precision (x axis) versus Recall (y axis) at the (b) family level, (d) genus level, and (f) species level. Metrics were averaged across all eight datasets.*

*Figure S2. Comparison of Metalign and competing methods, with no abundance cutoff, when applied to the challenge datasets from the first CAMI competition on the Weighted UniFrac metric, which is rank-independent (lower is better).*

* Metalign • Bracken_custom • Bracken_original

i Precision

i Recall

1

Fl score

Scaled Ll error

**C**

1 Precision

1

Fl score

Recall

Scaled Ll error

in

•

*

I

**a**

Metalign-

Ti)

>

a)

>, Bracken custom -

=	_

**E ca**

LI_

Bracken_original -

Metalign-

Ti.)

>

a)

0 Bracken custom -

D	_

c a)

(.7

Bracken_original -

1.0 **^b^**

0.8 -

0.6 -

0.2 -

0.0	I	I	I

0 0	0.2	0.4	0.6	0.8

Precision

**1.0 ^d^**

o

*

0.8 -

•

10

0.6 -

= co u a) *ec*

0.4 -

0.2 -

0.0	I	I	1

0 0	0.2	0.4	0.6	0.8

Precision

0.28

0.18

0.88

0.43

1 0

0.96

0.94

Metalign^-^

`,_:_f_)_ Bracken_custom - _ t.) a) a v^.^)

Bracken_original^-^

0.14

0.2

**f** 1.0

0.8 -

0.6 -

= ti3 u a) *cc*

0.4 -

0.2 -

0

*

**It**

1I	1 0.2	0.4	0.6	^1^

0.8

Precision

0.0

00

10

**e**

0.049

0.004

i

Precision

l	1

Recall	Fl score	Scaled

Ll error

0.66

**0.7**

*Figure S3. Comparison of Metalign Bracken+Kraken2 with the same custom database as Metalign, and Bracken+Kraken2 with its original database when applied to the challenge datasets from the first CAMI competition with no abundance threshold. Heatmaps show Precision, Recall, F1 score, and L1 error re-scaled such that higher is better (1 - (L1 error / 2)) at the (a) family level, (c) genus level, and (e) species level. Scatter plots show Precision (x axis) versus Recall (y axis) at the (b) family level, (d) genus level, and (f) species level. Metrics were averaged across all eight datasets.*

Metalign^-^

•

0.8 -

0.6 - =

a)

0.4 -

0.2 -

1.0

0.0

00	0.2	0.4	0.6	0.8	10

Precision

**a**

Metalign-	0.84

0.87	0.85

0.8

|  |  |  | 0.92 |
| --- | --- | --- | --- |
| >, Bracken custom  oz)  U- | 0.68 |  |  |

0.35

0.49

0.63

Bracken_original

0.89

Precision	Recall	Scaled

**Ll** error

0.63

| 0.9 |  |
| --- | --- |

**0.28**

I

**0.75**

**1.0**

7

*

0.8 -

0.6­= co u

a)

0.4 -

0.2 -

0.0

•

C

Metalign -	0.82	0.84	0.83

TL)

a)

in Bracken custom -

a)

(.7

Bracken_original -

0.19

|  | Recall |  | Scaled **Ll** error | 00 | 0.2 0.4 0.6 0.8  Precision | 10 |
| --- | --- | --- | --- | --- | --- | --- |
| Precision |  | Fl score |  |  |  |  |
|  |  |  |  |  |  |  |

0.013

0.13

**0.024**	0.039

_f_

1.0

0.8 -

m*

0.4 -

0.2 -

I

0.0

0.24

0.22

0.64

0.66

**0.15**

0.14

*av*

`,_:_f_)_ Bracken_custom -

a)
v^.^)

Bracken_original^-^

| Precision Recall Fl score | Scaled **Ll** error | 00 | 0.2 0.4 0.6 0.8  Precision | 10 |
| --- | --- | --- | --- | --- |

* Metalign • Bracken_custom • Bracken_original

*Figure S4. Comparison of Metalign, Bracken+Kraken2 with the same custom database as Metalign, and Bracken+Kraken2 with its original database when applied to the challenge datasets from the first CAMI competition with organisms below 0.01% relative abundance excluded. Heatmaps show Precision, Recall, F1 score, and L1 error re-scaled such that higher is better (1 - (L1 error / 2)) at the (a) family level, (c) genus level, and (e) species level. Scatter plots show Precision (x axis) versus Recall (y axis) at the (b) family level, (d) genus level, and (f) species level. Metrics were averaged across all eight datasets.*

*Figure S5. Comparison of Metalign, Kraken2 with the same custom database as Metalign, and Kraken2 with its original database when applied to the challenge datasets from the first CAMI competition in (a) wallclock running time, (b) CPU time, and (c) peak memory usage. The running times were summed across all eight datasets, while the memory values reflect the peak memory usage across all eight datasets. Due to computing resource limitations, Kraken2 with the custom database was run on a different server with more memory capacity and more compute threads, explaining its higher CPU time relative to wallclock time compared to its default database.*

**Second round of review**

**Reviewer 1**

Most of the issues I raised have been resolved. However, there are still some issues. Some of them are listed below.

Major revisions:
1.     Low figure quality issue. For example, font sizes of Figure 5 should be increased.
2.     The equations in the supplementary file are not shown correctly. Therefore, the definition for accuracy evaluation (such as precision, recall and F1 score) is not clear. Please double check.
3.     The information about computational environment, such operating system and hardware information, is not given for all the tools reported in the paper.
4.     The role of unclassified reads and their impact on the performance evaluation is not clear. In response to reviewer, authors showed that in the first CAMI high complexity benchmark database the majority (more than 70%) of the reads are classified as unknown. However, the impact of these majority number of reads on the performance (specificities, recalls et al) was not shown in the final results. This should at least be discussed as the disadvantage of alignment-based methods in the discussion section.

**Reviewer 2**

The authors have addressed most of majro comments. I only have two minor comments.

1) The manuscript originally focused on CAMI. Most discussions in Introduction are still around CAMI. It would be good discuss other evaluation papers to give a more complete overview of the status of metagenome classification.

2) It seems that the authors were only using 4 threads to run tools. For large datasets, we typically use more threads (e.g. 32 or even 64). I guess diamond can be well parallelized. How much wall-clock time would mega+dianmond take given more threads? Also relatedly, Fig. 3a and 5a should mention how many threads are used.

**Authors Response**

**Point-by-point responses to the reviewers’ comments:**

Comment

*Response*

Reviewer 1 Remarks to the Author:

Most of the issues I raised have been resolved. However, there are still some issues. Some of them are listed below.

Reviewer 1 Specific Comment #1:

Low figure quality issue. For example, font sizes of Figure 5 should be increased.

*Author Response:*

*We thank the reviewer for bringing our attention to this. We have increased the text size of all figures except Figures 4 and S2 (which already had rather large text). For instance, here is the new version of Figure 5:*

Reviewer 1 Specific Comment #2:

The equations in the supplementary file are not shown correctly. Therefore, the definition for accuracy evaluation (such as precision, recall and F1 score) is not clear. Please double check.

*Author Response:*

*We are very appreciative of the reviewer for pointing out this rendering error. The equations are now displayed as follows, with the same notation but proper rendering:*

<https://www.codecogs.com/eqnedit.php?latex=Precision%20%3D%20%5Cfrac%7BTP%7D%7BTP%2BFP%7D#0>

<https://www.codecogs.com/eqnedit.php?latex=Recall%20%3D%20%5Cfrac%7BTP%7D%7BTP%2BFN%7D#0>

<https://www.codecogs.com/eqnedit.php?latex=F1%20%3D%20%5Cfrac%7B2%20%5Ctimes%20Precision%20%5Ctimes%20Recall%7D%7BPrecision%20%2B%20Recall%7D#0>

<https://www.codecogs.com/eqnedit.php?latex=L1%20%3D%20%5Csum_%7Bi%3D1%7D%5ES%20%7C%20Predicted_i%20-%20Actual_i%20%7C#0>

Reviewer 1 Specific Comment #3:

The information about computational environment, such operating system and hardware information, is not given for all the tools reported in the paper.

*Author Response:*

*Thank you for bringing our attention to this omission. We have included a new section in our manuscript, entitled “Computing Environments” with this information. We also include it here:*

*Computing Environments*

*All experiments, with a few exceptions discussed next, were run on a server running Ubuntu 16.04.3 LTS (Xenial Xerus) with an 80 core Intel(R) Xeon(R) CPU E5-2698 v4 processor operating at 2.2 GHz and with 528 GB of RAM. A small number of experiments had to be run in different computing environments due to resource limitations when using the aforementioned server. Due to issues with memory usage, CLARK and Kraken2 (when trained on Metalign’s database) were run on a server running Ubuntu 18.04.4 with four AMD EPYC 7742 64-Core processors operating at 3.3GHz and with 1TB of RAM. Due to issues with running time, MEGAN+DIAMOND was run on the CAMI 1 data on a computing cluster, using various compute nodes with 64 GB of RAM.*

Reviewer 1 Specific Comment #4:

The role of unclassified reads and their impact on the performance evaluation is not clear. In response to reviewer, authors showed that in the first CAMI high complexity benchmark database the majority (more than 70%) of the reads are classified as unknown. However, the impact of these majority number of reads on the performance (specificities, recalls et al) was not shown in the final results. This should at least be discussed as the disadvantage of alignment-based methods in the discussion section.

*Author Response:*

*We thank the reviewer for bringing our attention to this. We have added a discussion of this issue to our discussion section, and mentioned the competing method MetaBinG2 which addresses this issue. Below is the new paragraph in the discussion section:*

*Finally, several additional analyses could be employed to evaluate Metalign’s performance more comprehensively, including comparisons with more methods and performance comparisons on other benchmark datasets such as those released by McIntyre et al* [*(McIntyre et al. 2017)*](https://paperpile.com/c/rrjTHH/NRvh) *and Ye et al* [*(Ye et al. 2019)*](https://paperpile.com/c/rrjTHH/hmhrq)*. All reference-based profiling methods will degrade in performance as the portion of organisms present in the sample but not in the reference database increases. This has been demonstrated empirically by Qiao et al* [*(Qiao et al. 2018)*](https://paperpile.com/c/rrjTHH/bZ98m) *using “clade exclusion experiments” to measure the relative performance degradation of different methods when taxonomic clades are excluded from the reference database. As Qiao et al showed that alignment-based methods can struggle in these settings relative to composition based methods like MetaBinG2* [*(Qiao et al. 2018)*](https://paperpile.com/c/rrjTHH/bZ98m)*, it would be interesting to measure how much Metalign’s performance degrades in such experiments relative to other methods.*

Reviewer 2 Remarks to the Author:

The authors have addressed most of major comments. I only have two minor comments.

Reviewer 2 Specific Comment #1:

TThe manuscript originally focused on CAMI. Most discussions in Introduction are still around CAMI. It would be good discuss other evaluation papers to give a more complete overview of the status of metagenome classification.

*Author response:*

*We appreciate this feedback, which helps us give a more complete picture of the field. We found that the McIntyre et al and Ye et al reviews discussed previously were largely in agreement with CAMI in that, for instance, MetaPhlAn2 was among the worst methods for precision and abundance estimation while Kraken was among the worst for precision. We have added the following red text to the third paragraph of the introduction to reflect this (with surrounding sentences included for context):*

*A recent widely-cited comprehensive benchmarking study, the Critical Assessment of Metagenome Interpretation (CAMI)* [*(Sczyrba et al. 2017)*](https://paperpile.com/c/rrjTHH/klHGu)*, evaluated ten widely-used profiling methods on a variety of simulated metagenomic datasets. Notably, the two profiling methods that performed best in precision (i.e., false positive rate) were the two methods ranked lowest for recall (i.e., false negative rate); similarly, the two best methods for recall were two of the three methods ranked lowest for precision* [*(Sczyrba et al. 2017)*](https://paperpile.com/c/rrjTHH/klHGu)*. Another major benchmarking study by McIntyre et al* [*(McIntyre et al. 2017)*](https://paperpile.com/c/rrjTHH/NRvh) *revealed similar patterns, with most non-alignment methods having either low precision and high recall or vise-versa. This makes sense intuitively as it is easy to select only a few high-confidence species as being present or to claim that almost all known species are present; conversely, balancing precision and recall presents a substantial challenge. This presents a difficult trade-off to researchers, who must choose between failing to identify most organisms or falsely identifying many organisms. The top-ranked method in L1-error and Weighted UniFrac* [*(C. A. Lozupone et al. 2007; C. Lozupone and Knight 2005)*](https://paperpile.com/c/rrjTHH/j8fCp+jVRG7)*, measures of error in abundance estimation and taxonomic composition, respectively, produced the second-worst precision* [*(Sczyrba et al. 2017)*](https://paperpile.com/c/rrjTHH/klHGu)*, indicating another difficult trade-off. Methods that ranked among the worst in recall and abundance estimation in the CAMI competition* [*(Sczyrba et al. 2017)*](https://paperpile.com/c/rrjTHH/klHGu) *and other benchmarking studies* [*(Ye et al. 2019; McIntyre et al. 2017)*](https://paperpile.com/c/rrjTHH/hmhrq+NRvh) *have been used in large-scale efforts to analyze human* [*(Human Microbiome Project Consortium 2012)*](https://paperpile.com/c/rrjTHH/yooDI) *and city metro* [*(Afshinnekoo et al. 2015)*](https://paperpile.com/c/rrjTHH/4QDu0) *microbiomes, and have also been used to perform downstream analyses that link the microbiome to host genetics* [*(Bonder et al. 2016)*](https://paperpile.com/c/rrjTHH/dS13r) *and diseases such as colorectal cancer, potentially impacting the findings of these important studies.*

*We also added a new paragraph to the discussion mentioning other benchmarking studies and also addressing comments from reviewer 1 about performance of alignment-based methods with incomplete reference databases. We hope this also helps add more perspective. Here is the new paragraph:*

*Finally, several additional analyses could be employed to evaluate Metalign’s performance more comprehensively, including comparisons with more methods and performance comparisons on other benchmark datasets such as those released by McIntyre et al* [*(McIntyre et al. 2017)*](https://paperpile.com/c/rrjTHH/NRvh) *and Ye et al* [*(Ye et al. 2019)*](https://paperpile.com/c/rrjTHH/hmhrq)*. All reference-based profiling methods will degrade in performance as the portion of organisms present in the sample but not in the reference database increases. This has been demonstrated empirically by Qiao et al* [*(Qiao et al. 2018)*](https://paperpile.com/c/rrjTHH/bZ98m) *using “clade exclusion experiments” to measure the relative performance degradation of different methods when taxonomic clades are excluded from the reference database. As Qiao et al showed that alignment-based methods can struggle in these settings relative to composition based methods like MetaBinG2* [*(Qiao et al. 2018)*](https://paperpile.com/c/rrjTHH/bZ98m)*, it would be interesting to measure how much Metalign’s performance degrades in such experiments relative to other methods.*

Reviewer 2 Specific Comment #2:

It seems that the authors were only using 4 threads to run tools. For large datasets, we typically use more threads (e.g. 32 or even 64). I guess diamond can be well parallelized. How much wall-clock time would megan+diamond take given more threads? Also relatedly, Fig. 3a and 5a should mention how many threads are used.

*Author Response:*

*We appreciate the reviewer drawing our attention to this. We have updated the captions for figures 3 and 5. We also added an additional experiment where we showed the scaling performance of MEGAN+DIAMOND versus Metalign when using 4, 8, 16, and 32 threads. The relevant text and figure are included here:*

*Finally, we assessed whether the discrepancy in runtime between MEGAN+DIAMOND and other methods could be reduced by increasing the number of threads used (Figure S6). We ran Metalign and MEGAN+DIAMOND on the first 5 million reads of the low complexity dataset from the first CAMI challenge with 4, 8, 16, and 32 threads. Both methods experienced a nearly linear speedup when using 8 threads instead of 4, but had diminishing improvements as the number of threads was scaled up to 16 and then 32. In all cases, Metalign was 20-50x faster than MEGAN+DIAMOND.*

*Figure S6. Comparison of Metalign and MEGAN+DIAMOND in terms of (a) wall clock time and (b) CPU time as they scale with the number of threads used (4, 8, 16, or 32). The dataset tested is the first 5 million reads of the low complexity dataset from the first CAMI challenge.*

**Third round of review**

**Reviewer 1**

All my comments have been addressed. I have no more comments.
